# Potential of histone deacetylase inhibitors in the control and regulation of prostate, breast and ovarian cancer

**DOI:** 10.3389/fchem.2022.948217

**Published:** 2022-08-12

**Authors:** Siddhartha Das Pramanik, Amit Kumar Halder, Ushmita Mukherjee, Dharmendra Kumar, Yadu Nandan Dey, Mogana R

**Affiliations:** ^1^ Department of Pharmaceutical Engineering and Technology, IIT-BHU, Varanasi, Uttar Pradesh, India; ^2^ Dr. B.C. Roy College of Pharmacy and Allied Health Sciences, Durgapur, West Bengal, India; ^3^ Department of Pharmaceutical Chemistry, Narayan Institute of Pharmacy, Gopal Narayan Singh University, Sasaram, Bihar, India; ^4^ Department of Pharmaceutical Biology, Faculty of Pharmaceutical Sciences, UCSI Education SDN.BHD., Kuala Lumpur, Malaysia

**Keywords:** HDAC inhibitors (HDACi), prostate cancer, breast cancer, ovarian cancer, Histone deacetylase (HDAC)

## Abstract

Histone deacetylases (HDACs) are enzymes that play a role in chromatin remodeling and epigenetics. They belong to a specific category of enzymes that eliminate the acetyl part of the histones’ -N-acetyl lysine, causing the histones to be wrapped compactly around DNA. Numerous biological processes rely on HDACs, including cell proliferation and differentiation, angiogenesis, metastasis, gene regulation, and transcription. Epigenetic changes, specifically increased expression and activity of HDACs, are commonly detected in cancer. As a result, HDACi could be used to develop anticancer drugs. Although preclinical outcomes with HDACs as monotherapy have been promising clinical trials have had mixed results and limited success. In both preclinical and clinical trials, however, combination therapy with different anticancer medicines has proved to have synergistic effects. Furthermore, these combinations improved efficacy, decreased tumor resistance to therapy, and decreased toxicity. In the present review, the detailed modes of action, classification of HDACs, and their correlation with different cancers like prostate, breast, and ovarian cancer were discussed. Further, the different cell signaling pathways and the structure-activity relationship and pharmaco-toxicological properties of the HDACi, and their synergistic effects with other anticancer drugs observed in recent preclinical and clinical studies used in combination therapy were discussed for prostate, breast, and ovarian cancer treatment.

## Introduction

Cancer is a significant public health problem worldwide and the second leading cause of death in the United States. An estimated 609,360 people in the United States will die from cancer in 2022, corresponding to almost 1700 deaths per day. The greatest number of deaths are from the cancers of lung, prostate, and colorectum in men and of the lung, breast, and colorectum in women (Siegel et al., 2022). According to the International Agency Global Cancer Burden (Ferlay et al., 2020), the number of new cases of cancer is 19.3 million whereas the death of cancer patients is 10 million in 2020. The most commonly diagnosed cancer, with an estimated 2.3 million new cases of female breast cancer (11.7%) has surpassed lung cancer (11.4%), colon and rectal (10%), prostate (7.3%), and stomach (5.6%) cancer. Lung cancer continues to exist as the most fatal type of cancer-causing millions of deaths (18%). HDACs are enzymes that play a role in chromatin remodeling and epigenetics. In cancers, HDACs are very often upregulated and can silence apoptosis-induced tumor suppressor genes to enhance the cancer progression (West and Johnstone, 2014). The active focus of several groups in the 1970s was the search for HDACs and proteins which have the potential of removing acetyl groups from histones. 18 types of mammalian HDACs were discovered so far and categorized into 4 major classes/groups. The first category or the class I of HDACs includes HDAC 1, 2, 3, and 8 while 4, 5, 7, and 9 are classified as class IIa type of HDACs. HDACs 6 and 10 are classified as class IIb while sirtuins (SIRT1-7) make up class III. HDAC 11 is included in the class IV type of HDAC (Seto and Yoshida, 2014). HDAC expressions that are abnormal are implicated in several phases of cancer and have become one of the symbols of hematological malignancy and tumors (Darwiche, 2020; Pant et al., 2020). HDACs are important players in cancer because they regulate a variety of cellular and molecular activities. HDAC inhibitors (HDACi) cause cell death by arresting the G1 phase of the cell cycle by reducing the cyclins and cyclin-dependent kinases (CDK) (Kim et al., 2000; Chun, 2015). Inhibition of HDACs also affects cancer cell apoptosis by controlling the expression of pro-and anti-apoptotic proteins (Zhang and Zhong, 2014; Gong et al., 2019). The discovery of particular HDACi has provided a valuable way to study HDACs biology. Hence, these inhibitors may act as a budding healing option for various chronic diseases like cancer, immunological and heart diseases (Losson et al., 2016).

The histone protein core is being wrapped throughout with the DNA in primitive eukaryotic cells, packaging, protecting, and regulating it. This structure, known as chromatin, is compressed and “closed,” which is linked to suppress the transcript process. On the other hand, it may be “open,” allowing transcription-control proteins to wrap DNA. Post-translational modifications (PTM) like phosphorylation, acetylation, methylation, and ubiquitination may play important roles in regulating chromatin in its many active states (Cui et al., 2014; Wang Y. C. et al., 2014). The well-understood mechanism is the reversible phosphorylation, acetylation, or methylation within histone tails to regulate chromatin. In many cells, the regulation of chromatin structure by histone PTMs has emerged as a significant driver of transcriptional responses. So, histone controls the protein machinery by the addition or deletion of the PTMs, which have proven pivotal in the understanding of physiological responses in a variety of cell types (Audia and Campbell, 2016).

Genetically, cancer is linked to the alteration in the genome and modifications, such as DNA methylation and histone alterations that can affect chromatin architecture. The nucleosome (the active part of chromatin) is wrapped around a histone core, which is made up of two core histones (H2A, H2B, H3, and H4) (Rhodes, 1997; Shaytan et al., 2015). Histone acetylation, phosphorylation, and methylation are the most prevalent epigenetic processes deregulated in cancer, where histone acetylation is having the most prominent role in cancer (Cheng et al., 2019; Rajan et al., 2020). Although several HDACi have been licensed for cancer treatment, clinical applications have been limited due to the HDACi’s poor pharmacokinetics, bioavailability, and selectivity, and most of them require the use of other medications to produce better outcomes. In the present review, efforts were made to evaluate the role of HDACi in the management of three common cancers: prostate, breast, and ovarian. Furthermore, a detailed discussion on the HDACi as the current medicines and clinical trials being conducted for the treatment of these cancers.

### HDACs: Classification, enzymatic activities, and biological function

Eighteen human HDACs have been found and classified into four groups based on their sequence similarity to yeast HDACs. HDACs 1, 2, 3, and 8 are all classified as class I HDACs. They are the most common type of HDACs found in the nucleus, and they are related to the yeast Rpd3 in several ways (Emiliani et al., 1998; Buggy et al., 2000; Hu et al., 2000). Class II HDACs are homologous to yeast Hda1 and greater in size than the other two classes. They are classified into two subgroups based on domain organization and sequence: class IIa and class IIb. The class IIa HDACs are inactive on acetylated substrates, thus differing from class I and IIb enzymes (Lahm et al., 2007). HDACs 6 and 10 belong to Class IIb, and they have an extra deacetylase domain (Miska et al., 1999; Kao et al., 2000; Zhou et al., 2001; Fischer et al., 2002; Guardiola and Yao, 2002; Kao et al., 2002). Class III, also known as sirtuins (SIRTs), is a group of proteins that includes SIRT1-7 and has a wide range of activity and location. SIRT1, 6, and 7 are mostly found in the nucleus, SIRT2 is mostly located in the cytoplasm, SIRT3, 4 and 5 mostly located in mitochondria (Lin et al., 2000; Carafa et al., 2012; Chen B. et al., 2015). Sirtuins are NAD+ -dependent class III protein deacetylases that can be found in anything from bacteria to humans (Kida and Goligorsky, 2016). Sirtuins can sense changes in cellular energy by utilizing NAD+ as a cofactor for their enzymatic activity. Cell survival during stress, metabolic balance, chromatin control, and cell differentiation are the activities that Sirtuins can accomplish (Pant et al., 2017a; Wang Y. et al., 2019). Only HDAC 11 are found in Class IV (Gao et al., 2002). SIRTs are NAD+ dependent enzymes, whereas the other three types are Zinc cation (or Zn2+ ion)-dependent HDACs (Imai and Guarente, 2014). The classification, locations, and diverse enzymatic activities of HDACs are shown in Table 1. The IC50 values of each HDACIs have been shown in Table 2.

**TABLE 1:** Classification of HDACs.

Sl no.	Class	Member	Enzymatic activities	Subcellular localization
1	HDAC I	HDAC 1	Deacetylase, decrotonylase, O-GlcNAcylation	Nucleus
		HDAC 2	Deacetylase, S-nitrosylase	Nucleus
		HDAC 3	Deacetylase, decrotonylase	Nucleus
		HDAC 8	Fatty acid deacylase	Nucleus/cytoplasm
2	HDAC II	HDAC4	Deacetylase, O-GlcNAcylation	Nucleus/cytoplasm
		HDAC5	Deacetylase	Nucleus/cytoplasm
		HDAC6	Deacetylase, O-GlcNAcylation, fatty acid deacylase	Mostly cytoplasm
		HDAC7	Deacetylase	Nucleus/cytoplasm/mitochondria
		HDAC9	Deacetylase	Nucleus/cytoplasm
		HDAC10	Polyamine deacetylase	Mostly cytoplasm
3	HDAC III	SIRT 1	ADP-ribosyltransferase, deacetylase, S-glutathionylase, O-GlcNAcylation	Nucleus/cytoplasm/mitochondria
		SIRT 2	ADP-ribosyltransferase, benzoylase	Nucleus/cytoplasm/mitochondria
		SIRT 3	ADP-ribosyltransferase, deacetylase	Nucleus/cytoplasm/mitochondria
		SIRT4	ADP-ribosyltransferase, de-methylglutarylase, lipoamidase, de-hydroxymethylglutarylase, de-3-methylglutaconylase	Mitochondria
		SIRT5	ADP-ribosyltransferase, desuccinylase, demalonylase	Nucleus/cytoplasm/mitochondria
		SIRT6	ADP-ribosyltransferase, fatty acid deacylase	Nucleus/cytoplasm
		SIRT7	ADP-ribosyltransferase, desuccinylase, deglutarylase	Nucleus/cytoplasm
4	HDAC IV	HDAC11	Fatty acid deacylase	Nucleus/cytoplasm

**TABLE 2:** IC_50_ values of HDACs inhibition.

HDACs class	HDACs isozyme	Romidepsin	Panobinostat	Belinostat	Vorinostat	Tucidinostat
Class I	HDAC1	1 nM	3 nM	26 nM	60 nM	0.1 μM
	HDAC2	1 nM	2 nM	22 nM	42 nM	0.2 μM
	HDAC3	1 nM	2 nM	19 nM	36 nM	0.1 μM
	HDAC8	>1,000 nM	22 nM	22 nM	173 nM	0.7 μM
Class II	HDAC4	647 nM	1 nM	15 nM	20 nM	>10 μM
	HDAC5	>1,000 nM	1 nM	25 nM	36 nM	>10 μM
	HDAC6	226 nM	1 nM	10 nM	29 nM	>10 μM
	HDAC7	>1,000 nM	2 nM	51 nM	129 nM	>10 μM
	HDAC9	>1,000 nM	1 nM	24 nM	49 nM	>10 μM
	HDAC10	1 nM	31 nM	59 nM	60 nM	0.1 μM
Class IV	HDAC11	0.3 nM	4 nM	27 nM	31 nM	0.4 μM

### Histone deacetylation and cancer initiation

Through their effects on chromatin compaction and the stability of other cellular target proteins, HDACs play a critical role in the epigenetic regulation of gene transcription and expression (Ropero and Esteller, 2007). Dysregulation of DNA methylation and post-translational histone modifications, particularly histone acetylation, are common features of human cancer, with the disastrous result of gene transcription deregulation. Loss of acetylated Lys16 (K16-H4) and trimethylated Lys20 (K20-H4) of histone H4 is linked to hypomethylation of repetitive sequences and is a common event in human cancer (Fraga et al., 2005). Another research on gastrointestinal malignancies found that the decreased histone acetylation is linked to tumor invasion and metastasis as well as tumorigenesis (Yasui et al., 2003). HDACs overexpression has been shown in a variety of solid and hematological malignancies (Shankar and Srivastava, 2008), influencing a variety of cellular functions including proliferation, cell death, metastasis, autophagy, metabolism, and ciliary expression.

HDACs have been shown to regulate apoptosis in cancer cells by changing the expression of apoptotic proteins. HDAC2 expression was found to be abnormal in cancer cells, and HDAC2 inhibitors suppressed cell motility, invasion, and proliferation, as well as causing cell death, in gastric cancer cells (Kim et al., 2013). In human lung cancer cells, inhibiting HDAC2 inhibition leads to the activation of p53 and Bax that inhibits tumor cell proliferation causing cell death (Jung et al., 2012). HDACs are involved in the deacetylation of non-histone proteins as well as histone deacetylation, hence their role in cancer is multifaceted. *In vivo* and *in vitro*, HDAC1 interacts with the tumor suppressor p53 and deacetylates it (Juan et al., 2000; Luo et al., 2000). Under stressful situations, p53 is phosphorylated and acetylated. Since acetylated lysine residues in p53 overlap with ubiquitinated lysine residues, p53 acetylation promotes protein stability and activation, prompting cell-division checkpoints, persistent cell-division arrest, and cell death.

HDAC2 was also discovered to favorably control Aurora A kinase, which promotes pancreatic cancer cell proliferation while inhibiting cell death by inducing ciliary loss (Kobayashi et al., 2017). p53 is a tumor suppressor gene that can cause altered cells to die. The effect of p53 acetylation in tumor suppression has been extensively studied (Dai and Gu, 2010). MDM2 ubiquitinates p53 and leads to its destruction when HDACs such as SIRT1 and SIRT2 remove acetyl groups from p53’s C-terminal lysines, reducing p53 levels in cells (Lee and Gu, 2013). Lower p53 levels in cells may promote cell proliferation while inhibiting apoptosis.

SIRT1 controls histone acetylation (mostly at the K16-H4 and K9-H3 locations) and the acetylation of transcription factors such as p53 (Vaziri et al., 2001), p300 histone acetyltransferase (Bouras et al., 2005), E2F1 (Wang et al., 2006), DNA repair ku70 (Cohen et al., 2004; Fu et al., 2006). When all of these factors are considered, it is obvious that sirtuin dysregulation plays a role in cancer development. SIRT1 is upregulated in lung cancer (Yeung et al., 2004), prostate cancer (Kuzmichev et al., 2005), and leukemia (Bradbury et al., 2005), but downregulated in colon cancer (Özdağ et al., 2006).

When it comes to the role of HDACs in cancer, there are several mechanisms through which HDACs contribute to cancer development. The majority of investigations to date have focused on the impact of abnormal HDACs recruitment to certain promoters *via* interactions with fusion proteins resulting from chromosomal translocations common in hematological malignancies. Acute promyelocytic leukemia (APL) is an archetypal case that serves as a paradigm for various other hematological cancers. The chromosomal translocation that results in fusion proteins containing RAR-PML and RARPLZF is a hereditary feature of this disease. These fusion proteins bind to retinoic acid-responsive elements (RAREs) and recruit the HDACs repressor complex with high affinity, blocking retinoic acid-binding and suppressing the expression of genes that control myeloid cell differentiation and proliferation (Lin et al., 2001).

HDACs are known to deacetylate a wide range of proteins, including those involved in cell cycle regulation. The S phase and M phase transitions are critical for genomic integrity (Telles and Seto, 2012). The E2F members interact with retinoblastoma protein (pRb) to cause cell cycle advancement and apoptosis. By attracting HDAC 1 to E2F-responsive promoters, pRb can inhibit the E2F-mediated transcription of cell cycle proteins (Brehm et al., 1998).

CDH1, or epithelial-cadherin, is a cell marker that is decreased during metastasis. HDAC1 is recruited to the CDH1 promoter in pancreatic cancer cells, resulting in deacetylation of histone 3 and 4 proteins in the nucleus and E-cadherin depletion, which aids in the epithelial-mesenchymal transition (EMT) (Von Burstin et al., 2009). To suppress E-cadherin expression, the transcription factor Snail binds HDAC1, HDAC2, and the corepressor complex mSin3A to the promoter (Peinado et al., 2004). Downregulation or loss of function of E-cadherin has been linked to carcinomas gaining invasive capacity (Christofori and Semb, 1999; Hajra and Fearon, 2002), suggesting that abnormal recruitment of HDACs to this promoter could play a role in tumor invasion and metastasis.

HDACs activity may be controlled directly by several metabolites produced by various intracellular metabolic processes. The addition of NADPH and Coenzyme A to recombinant HDAC1 and HDAC2 complexes boosted their cellular activity (Vogelauer et al., 2012). A bioactive lipid sphingosine-1 phosphate, generated during nuclear sphingolipid metabolism involved in the oxidation of fatty acids, was discovered to suppress HDAC activity by binding to its active site, leading to more research into the function of metabolic control in HDAC activity (Hait et al., 2009; Huang et al., 2012). HDAC3 has oncogenic effects in cholangiocarcinoma (CCA) cells, according to Yin et al. (2017), by suppressing apoptosis and promoting cell growth. Furthermore, elevated HDAC3 and HDAC6 expressions were found in CCA patients’ tissues, which were associated with a poor prognosis (Hubbert et al., 2002; Gradilone et al., 2013; Yin et al., 2017).

The role of autophagy in the genesis, maintenance, and advancement of cancer cells has been intensively researched. HDACs like HDAC6 (Gradilone et al., 2013) and HDAC10 deacetylate cytoplasmic proteins in CCA cells, and they’ve been linked to the autophagy process through modulating critical autophagy proteins including LC3-II and Beclin1 (Koeneke et al., 2015). The increased autophagic flow was seen in cells lacking class I HDACs, as evidenced by increased autophagosomal proteins such as LC3-II, Beclin1, and ATG5 (Schipper et al., 2014). Higher expression of autophagy regulators involved in various cell functions is linked to the depletion of HDACs such as class I and IIa isozymes as autophagy can promote cancer cell survival, simultaneously targeting autophagy might improve the therapeutic effects of HDACi against cancer.

### HDACi in cancer therapy

HDACi have shown potent therapeutic effects in different types of cancers, in both preclinical research and clinical trials. HDACi can considerably reduce cancer burden by attenuating tumor growth and regulating aberrantly proliferating vasculature (Guerriero et al., 2017). HDACi can suppress HDAC activity, promote histone acetylation aggregation in autosomes, and induce gene expression (Sanaei and Kavoosi, 2019). HDAC is have been found to have anticancer effects in cancer cells by a variety of pathways, including cell cycle arrest, apoptosis, and autophagy induction (Lam et al., 2013; Luchenko et al., 2014). Sodium butyrate was discovered in the 1970s to be able to transform red leukemia cells into normal cells and resynthesize haemoglobin. The first HDACi was discovered as a result of this process, which was accompanied by severe histone hyperacetylation (Ginsburg et al., 1973; Riggs et al., 1977).

Following FDA approval, HDACi are currently divided into four categories: (i) hydroxamic acids or hydroxamates, such as e.g., trichostatin A (TSA) and vorinostat, panobinostat and belinostat; (ii) cyclic peptides, including depsipeptides, tetrapeptides [e.g., romidepsin (FK228)]; (iii) benzamides, such as chidamide, entinostat (MS-275); and (iv) short-chain fatty acids, including valproic acid (VPA) and phenylbutyrate (Li and Zhu, 2014; Li and Seto, 2016). Tsuji et al. (1976) isolated the first natural HDACi, TSA, which was derived from *Streptomyces hygroscopicus*. Following the discovery of TSA, trapoxin was isolated from fungi and also found to act as an HDACi (Itazaki et al., 1990).

HDACi can also increase cancer cell death by inducing DNA damage, cell cycle arrest, apoptosis, and autophagy, as discussed previously. Some new SIRT inhibitors, such as MC2494, MHY2245, MHY2256, tenovin-6, and YC8-02, mediate apoptosis or autophagy, and so have anti-tumor properties (De et al., 2018; Tae et al., 2018; Li et al., 2019; Igase et al., 2020). The hydroxamic acid class includes vorinostat, the first FDA approved HDACi to treat patients with cutaneous T-cell lymphoma (CTCL). In 2009, the FDA approved the cyclic peptide romidepsin for the treatment of CTCL. In 2014, the FDA approved panobinostat and belinostat for the treatment of peripheral T-cell lymphoma (PTCL), with belinostat receiving additional clearance from the European Medicines Agency (EMA) (Andreu Vieyra and Berenson, 2014). The majority of SIRT inhibitors are still in the preclinical stage. Only nicotinamide (vitamin B3) has been used in clinical trials to treat cancer thus far (e.g., NCT02416739 and NCT00033436). Nicotinamide has been found to have a function in the prevention of nonmelanoma skin cancers, which are caused mostly by UV exposure (Chen et al., 2015).

Natural substances offer a wide spectrum of medications that are both powerful and pleiotropic. Various HDACi have so far been discovered to be of natural origin. HDACi like FK322, a cyclic peptide derived from *Chromobacterium violaceum*, and TSA from *S. hygroscopicus* inhibit HDAC 1 and 2 activities selectively. HDACi derived from a fungus, trapoxin A and depudecin, are also naturally occurring HDACi. Natural HDACi, such as largazole and azumamides, are also found in some marine organisms (Bassett and Barnett, 2014; Singh et al., 2018). Butyrate is a short-chain fatty acid produced by the gut microbiota during the fermentation of dietary fibers, and it has been found to inhibit HDACs class III, which affects post-translational modifications of histones (Delage and Dashwood, 2008; Morrison and Preston, 2016). Butyrate has been shown to reduce the growth of hepatocellular carcinoma (Wang et al., 2013; Pant et al., 2017b), lung cancer (Amoêdo et al., 2011), breast cancer (Chopin et al., 2004), pancreatic cancer (Farrow et al., 2003; Natoni et al., 2005), and colon cancer cells by increasing histone acetylation (Donohoe et al., 2012, 2014).

HDACi are currently useful for treating a variety of cancers, although adverse effects such as diarrhea, myelosuppression, and cardiovascular toxicity limit their use. Because HDACs affect various cellular pathways and current HDACi are mainly non-isoform-selective, several adverse effects are possible (pan-HDACi). As a result, more potent and isoform-selective HDACi are still needed like SB-429201, PCI-34051, SB-379278A, tubacin (Bieliauskas and Pflum, 2008).

### Prostate cancer

Prostate cancer is the most commonly diagnosed cancer in men and the second greatest cause of death from cancer. Prostate cancer is the most frequent cancer among males in the United States, with over 34,000 men died from it in 2021. (Siegel et al., 2022). It is also worth noting that it is the second-leading cause of cancer death among men (Kaushik et al., 2015). Even while localized prostate cancer has a 100%, 5-year survival rate, it reduces to 29.3 percent when cancer spreads to other organs (Damodaran et al., 2017). Furthermore, between 2005 and 2018, the number of new cases of prostate cancer increased by 31%, from 9,74,000 to 1.3 million (Fitzmaurice et al., 2017; Rawla, 2019). After the initial diagnosis, most patients are treated with localized radical prostatectomy, radiation therapy, proton beam therapy, and cryosurgery (Hayden et al., 2010; Ukimura, 2010). Patients with metastatic illness or recurring cancer with the localized region and distant metastases, should consider androgen deprivation therapy (ADT) or castration therapy as the first line of treatment (Perlmutter and Lepor, 2007). Unfortunately, despite an excellent initial therapeutic response, most patients treated with ADT eventually develop a very aggressive and therapy-resistant type of prostate cancer, resulting in poor clinical outcomes (Miller et al., 2017; Moreira et al., 2017).

The human prostate is a walnut-sized glandular structure that emerges from the urogenital sinus during embryonic development (Lee et al., 2011). Its main job is to make seminal fluid, which contains zinc, citric acid, and a variety of enzymes, including prostate-specific antigen protease (PSA). Because of the lack of well-characterized prostate epithelial lineage, the cellular origin of prostate cancer is unclear (Stoyanova et al., 2013; Wang et al., 2014). Because prostate cancer cells rely on androgens for survival in the early stages, androgen removal is the most common systemic treatment, which is hypothesized to act by inducing apoptosis (Isaacs, 1994; Montironi et al., 1998). Androgen deprivation therapy has been the standard of care for advanced prostate cancer, resulting in remission in 80%–90% of men with advanced disease and a median disease-free survival of 12–33 months. Unfortunately, despite a past response to androgen deprivation, neoplastic cells will continue to proliferate in the majority of individuals. Castration-resistant prostate cancer (CRPC) is a progressive form of prostate cancer with a median overall survival of 23–37 months after the onset of androgen deprivation (Hellerstedt and Pienta, 2002). Patients with CRPC who are unresponsive to androgen deprivation and/or chemotherapy require newer therapeutic agents. By improving the binding between histones and the DNA backbone, the HDACs family of enzymes limits the expression of genomic regions.

### Development HDACi against prostate carcinoma

**FIGURE 1:**
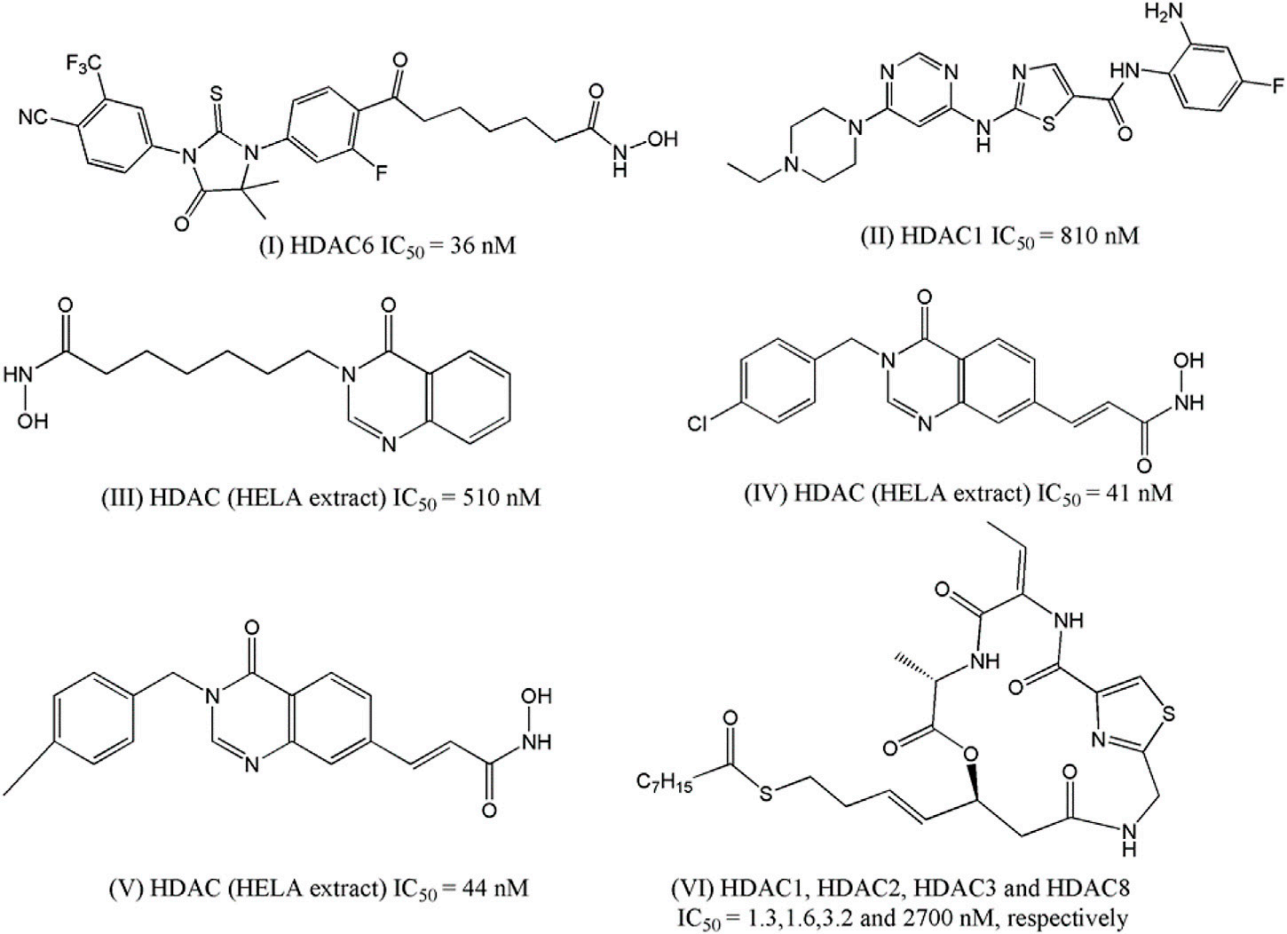
Structures of HDACi designed in recent years depicted anti-proliferative potential against the prostate carcinoma cell lines.

Jadhavar et al. (2016) designed some androgen receptor (AR)-HDAC6 inhibitors based on the structures of AR inhibitor enzalutamide and HDACi vorinostat and panobinostat. After synthesizing fourteen hybrid compounds, a few compounds were selected based on inhibitory potential against a range of HDACs (HDAC-1, 2, 3, and 6) and AR as well as on selectivity towards HDAC6 as compared to other HDACs. For example, one compound, the structure of which is presented in Figure 1 as (I), has an IC_50_ value of 36 nM against HDAC6 whereas its inhibitory potency against other HDACs (e.g., HDAC1, HDAC2, and HDAC3) is found in the micromolar range. HDAC6 has been reported to be involved in the acetylation of heat shock protein 90 (Hsp90) to regulate the nuclear localization and activation of the AR. Therefore, dual HDAC6/AR inhibitors are likely to produce synergistic effects that may conveniently be utilized as a new approach for the treatment of prostate cancer. The cytotoxicity of some dual HDAC6/AR inhibitors was therefore tested by these investigators with breast carcinoma cell line MDA-kb2 to establish that the designed compounds have similar cytotoxic potential as enzalutamide. When tested on prostate cancer cell line LNCaP, hyperacetylation of tubulin was obtained from two compounds.

Chen and co-workers (Chen et al., 2016) attempted to develop compounds with dual Bcr-Abl and HDACi by combining the fragment of dasatinib (one well-known clinically used Bcr-Abl inhibitor) with the zinc-binding domain of HDACi named MS-275. The designed structure lacked hydroxylamine moiety and instead consisted of o-Phenylenediamine residue that served as a zinc-binding domain in the HDACs. The synthesized compounds were first tested against Bcr-Abl and HDAC-1. The most potent compounds were then subjected to enzymatic assay against multiple HDACs enzymes and their antiproliferative potential was measured against three cell lines including prostate cancer cell line DU145. The most potent compound (II, Figure 1) was found to have an IC_50_ of 0.60 µM against this cell line and at the same time, it also showed micromolar potencies against the HDAC-1 enzyme (IC_50_: 0.81 µM).

Hieu et al. (2018) developed quinazolin-4(3H)- one based HDACi on the basis of the fact that this moiety is frequently found in various therapeutic agents. In their designed compounds, this moiety was assumed as a cap group whereas either N-hydroxybenzamides or N-hydroxypropenamides was used as zinc binding group. Sixteen synthesized compounds were tested against HDACs as well as against three different cancer cell lines including prostate cancer cell line PC-3. The most active HDACi was reported with IC_50_ value of 90 nM whereas the same compound depicted an IC_50_ of 810 nM against PC-3 cell. Considering that both these activities are higher than standard HDACi vorinostat, it may be inferred that the design was indeed successful, as far as the efficacy is concerned. By modifying the linker moiety of these structures, the same group reported another investigation (Huan et al. (2019), and this time too, the biological activities of some synthesized compounds were found to be highly satisfactory as these both HDACi as well as cytotoxic agent. For example, one compound, structure of which is presented in Figure 1 as III, depicted IC_50_ of 520 nM against HDACs (HELA extract) whereas the IC_50_ value of 210 nM was obtained in the cytotoxicity assay against PC-3 cell line. Noticeably, the cytotoxic potency of this compound was 15-times higher than that of vorinostat. More recently, another library of compounds was designed and synthesized by this group keeping the quinazolin-4(3H)-one moiety intact but generating various derivatives by changing substituents at the N-3 position of this aromatic moiety as well as by varying the zinc binder groups (Anh et al., 2021). Similar to earlier investigations, these compounds were tested against multiple cancer cell lines and one of these was PC-3. Two compounds (IV and V of Figure 1) were found to be the most potent derivatives. Their HDAC inhibitory activity (IC_50_ values, 0.041–0.044 μM) and cytotoxicity (IC_50_ values, 0.671–1.211 μM) were even higher than those of vorinostat. Furthermore, some compounds showed up to 10-fold more potent HDAC6 inhibition compared to their inhibitory activity in total HDACs extract assay. Further analyses of IV and V revealed that these compounds strongly induced both early and late apoptosis and arrested SW620 cells at the G2/M phase.

Zhang et al. (2020) took a different approach to successfully design HDACi by modifying the structure of romidepsin, which is a clinically approved HDACi used for the treatment of lymphomas. The investigators initially identified a novel cyclic depsipeptide through structural modification of romidepsin and this novel compound was found to selectively block class I HDACs. A series of novel cyclic depsipeptides was then prepared and screened against HDACs to select the most potent derivatives. On one hand, these compounds selectively inhibited class I HDACs and at the same time displayed nanomolar antiproliferative potencies against a range of cancer cell lines, and one compound (VI, Figure 1) was reported with GI_50_ of 7 nM against prostate cancer cell PC-3 and more importantly it showed 200 times more selectivity against human normal cell line.

### HDACs and prostate cancer

The molecular processes governing prostate cancer cell proliferation in an androgen-deficient environment are now being investigated. Covalent acetylation and deacetylation of histone proteins are one of these methods. The transcription of proto-oncogenes and tumor suppressor genes is regulated by these covalent changes. In prostate cells, HDACs regulate the expression of various functional genes, including the androgen receptor (AR). As a result, HDACi are currently being studied in CRPC patients. It has recently been discovered that the majority of recurrent prostate tumors are dependent on the AR signaling axis rather than being hormone-refractory or androgen-independent (Montgomery et al., 2008). The AR is a cytoplasmic protein that binds to testosterone or dihydrotestosterone in the nucleus, causing gene transcription to change. The synthesis of intracrine androgens may be important in sustaining tumoral androgen levels and the progression of CRPC (Stanbrough et al., 2006). HDACs are widely produced and elevated in prostate cancer, according to several lines of evidence (Waltregny et al., 2004; Weichert et al., 2008). Using a patient cohort of 192 patients who underwent radical prostatectomy, Weichert and colleagues investigated the expression patterns of HDACs 1, 2, and 3 in prostate cancer. HDAC 1 and 2 were found to have a positive correlation with Gleason scores, with high-grade tumors expressing both isoforms at higher rates. Furthermore, the Ki-67-positive proliferative fraction of prostate cancer cells is linked substantially with HDACs 1, 2, and 3, indicating increased cellular proliferation (Weichert et al., 2008).

During the malignant transformation of prostate epithelial cells, hypermethylation of CpG islands and chromatin remodeling play essential roles in the suppression of several tumor suppressor genes. It has been shown that DNMT1 and HDAC1 levels are higher in prostate cancer than in BPH, implying that they play a role in DNA-methylation-induced chromatin remodeling-induced inactivation of different essential genes (Patra et al., 2001). Dihydrotestosterone causes acetylation of the androgen receptor (AR), and HDACi increase p300 binding while reducing N-CoR/HDAC/Smad3 co-repressor binding, improving cell survival and growth in prostate cancer cells both *in vivo* and *in vitro* (Fu et al., 2003). NAD-dependent SIRT1 is necessary for androgen antagonist-mediated transcriptional repression and growth suppression, according to new research. SIRT1 and nuclear receptor co-repressor are recruited to AR-responsive promoters by androgen antagonist-bound androgen receptor, which deacetylates histone H3 locally at the PSA promoter (Dai et al., 2007). ARR19 is a new AR co-repressor that engages HDAC4, resulting in AR transactivation suppression (Jeong et al., 2004). Androgen has also been shown to play a role in the nuclear localization of HDAC4 and to be more prevalent in the nucleus in more aggressive prostate tumors (Halkidou et al., 2004). These data show that androgen, HDAC4, and ARR19 all play key roles in the progression of prostate cancer. Maspin, a tumor suppressor, has been linked to malignancies that are better differentiated, more responsive to pharmacological therapy, and have a better prognosis. In human prostate cancer cell lines and tissues, Maspin interacts with HDAC1 (Li et al., 2006). HDAC1 is inhibited by this direct molecular interaction in both the nucleus and the cytoplasm *via* glutathione S-transferase (GST), according to studies (Li et al., 2006). Through DNA methylation and chromatin changes, the hDAB2IP gene is epigenetically repressed in prostate cancer (Chen et al., 2003). Ezh2, a histone lysine methyltransferase, and HDAC I are involved in the downregulation of hDAB2IP (Chen et al., 2005). HDACIs with anti-prostate cancer activities have been shown in Table 3.

**TABLE 3:** | HDACi with anti-prostate cancer activities.

Sl no.	Compounds	*In vitro* activity	*In vivo* activity
01	CUDC-907	Inhibition of HDACs and PI3K, apoptosis induction, increased Bim, suppressed Mcl-1 and Bcl-xL	Tumor growth inhibition by 60% without weight loss (LuCaP 35CR patient-derived mouse xenografts)
02	CN133	Inhibition of HDAC1-3; 100 times more active than vorinostat (22Rv1 cells), inhibition of cell migration, invasion and AR signaling	Tumor growth and weight reduction by 50% (22Rv1)
03	3BrQuin-vorinostat, 3ClQuin-vorinostat	Higher antiproliferative activity than gefitinib (DU145 cells), HDAC inhibition, EGFR inhibition, mTOR suppression	NA
04	CUDC-101	Suppressed AR, AR-V7, and HER2	Significant tumor growth inhibition without weight loss (22Rv1)
05	2–75	HDAC inhibitory activity, induced p21, higher acetyl-tubulin levels (based on stronger HDAC6 inhibition) than vorinostat, suppressed Hsp90 and AR/AR-V7	Improved long-term tumor growth inhibition, enhanced apoptosis, reduced nuclear AR accumulation (LNCaP)

### Preclinical studies of HDACi in prostate cancer

Inhibition of Class I HDAC1, −2, and −3 by the adamantyl-capped HDACI CN133 was superior to vorinostat (IC_50_ = 0.6, 2, and 0.3 nM for CN133; 4, 11, and 3 nM for vorinostat), whereas vorinostat was more potent against HDAC6 (IC_50_ = 2 nM) than CN133 (IC_50_ = 4.1 nM). In 22Rv1 CRPC cells, CN133 was 100 times more antiproliferative (IC_50_ = 10 nM) than vorinostat (IC_50_ = 1 M). CN133 reduced AR signaling and decreased CRPC cell migration and invasion. Mice with 22Rv1 CRPC were given CN133 (1 mg/kg), which reduced tumor volume and weight by 50% when compared to placebo-treated mice (Chen et al., 2021).

Compound 2–75 is an enzalutamide hybrid with HDAC inhibitory activity that promoted p21, increased acetyl-tubulin levels (due to enhanced HDAC6 inhibition), and lowered Hsp90 and AR protein levels in C4-2 prostate cancer cells (Rosati et al., 2016). Deeper studies of 2–75 in CRPC were conducted based on these findings. DHT-induced AR transcriptional activity and AR translocation to the nucleus were reduced by compound 2–75 more effectively than enzalutamide. In addition to AR, 2–75 downregulated the mutant AR-V7 in prostate cancer cells in a proteasome-dependent manner, implying that 2–75-treated cells had better AR breakdown. *In vivo* tests with LNCaP tumor models demonstrated that 2–75 treatment (10 mg/kg, intratumoral injection twice weekly) exhibited tumor growth inhibitory effect comparable to enzalutamide, but that 2–75 had better tumor growth suppression in the long run (after Day 24) when compared to enzalutamide. In the tumor bodies of treated animals, 2–75 activity was associated with enhanced apoptotic induction and decreased AR nuclear accumulation (Hu et al., 2019).

Compound CUDC-101 suppressed HDACs, EGFR, and HER2 by combining an HDAC inhibitory fragment with an EGFR inhibitory scaffold derived from the authorized anticancer active EGFR inhibitor erlotinib (Lai et al., 2010). CUDC-101 inhibited full-length AR as well as the mutant AR-V7 form in CRPC cells, increased p21, and decreased HER2/NEU. CUDC-101 (50 g/kg/day for 14 days) effectively suppressed tumor growth in castrated mice with aggressive 22Rv1 CRPC tumors, with no discernible weight loss in the treated mice (Sun et al., 2016). Erlotinib and CUDC-101 both have limitations that necessitate more research. Cytochrome P450 enzymes can activate the ethinylphenyl residue of erlotinib, resulting in oxidized phenol and quinone molecules with toxicity potential (Li et al., 2010). Indeed, in cancer patients receiving corticosteroids or ciprofloxacin the erlotinib has been shown to increase the incidence of deadly gastrointestinal tract perforations (Gass-Jégu et al., 2016).

In DU145 CRPC cells, the chimeric compounds 3ClQuin-vorinostat and 3BrQuin-vorinostat showed 3–4 times stronger growth inhibitory action (IC_50_ = 3.23 M for 3ClQuin-vorinostat and 3.53 M for 3BrQuin-vorinostat) than gefitinib (IC_50_ = 11.9 M); however, vorinostat (IC_50_ = 0.68 M) was still more antiproliferative. Nonetheless, 3ClQuin-vorinostat and 3BrQuin-vorinostat combined EGFR inhibitory efficacy with HDAC inhibition, decreased EGFR expression in DU145 cells to a level comparable to vorinostat, showed only minor unspecific toxicity, triggered death, and prevented angiogenesis (Goehringer et al., 2021). As a result of the reduced erlotinib (and vorinostat) toxicity and resistance generation, these chimeric compounds may be suitable anticancer therapeutic candidates in prostate cancer therapy.

Another promising HDAC/kinase inhibitor, CUDC-907 (fimepinostat), was developed to target HDAC enzymes as well as the kinase PI3K (Qian et al., 2012). CUDC-907 decreased HDACs and PI3K signaling in a panel of eight prostate cancer cell lines, promoted apoptosis in a dose-dependent manner linked with enhanced pro-apoptotic Bim, and lowered antiapoptotic Mcl-1 and Bcl-xL expression in 22Rv1 CRPC cells, and inhibited HDACs and PI3K signaling. Furthermore, CUDC-907 treatment increased DNA damage because DNA damage response proteins were downregulated (Wee1, CHK1, RRM1, and RRM2). Finally, in castration-resistant LuCaP 35CR mice xenografts, CUDC-907 (100 mg/kg/day, p.o.) suppressed *in vivo* tumor growth by around 60% while causing little weight loss (Hu et al., 2020).

### Registered HDACi in clinical trials of prostate cancer

Several clinical trials with HDACi have been conducted to assess the benefits and drawbacks of their use in clinics. Only the most important findings from clinical trials with HDACi for the treatment of castration-resistant prostate cancer conditions are reported in Table 4.

**TABLE 4:** HDACi in a clinical trial for the treatment of prostate cancer.

Sl no.	Drug	Combination	Phase	Cancer types	References
1	Panobinostat	Bicalutamide	2	Metastatic castration-resistant prostate cancer	NCT00878436 Ferrari et al. (2019)
2	MS-275	Enzalutamide	1	Castration-resistant prostate cancer	NCT03829930 Lin et al. (2021)
3	Valproic acid	Bevacizumab	1	Metastatic castration-resistant prostate cancer	NCT00530907 Wheler et al. (2014)
4	Vorinostat	Euprolide acetate	2		NCT00589472 Cha and Eastham, (2015)
5	Panobinostat		2	Metastatic castration-resistant prostate cancer	NCT00667862 Rathkopf et al. (2013)
6	Azacitidine	Docetaxel/prednisone	1,2	Metastatic Prostate Cancer	NCT00503984 Singal et al. (2015)
7	Vorinostat		2	Metastatic Prostate Cancer	NCT00330161 Bradley et al. (2009)
8	Vorinostat	Temsirolimus	1	Metastatic Prostate Cancer	NCT01174199 Park et al. (2016)
9	Panobinostat		2	Metastatic Hormone Refractory Prostate Cancer	NCT00667862 Rathkopf et al. (2013)
10	Panobinostat	Bicalutamide	1,2	Recurrent Prostate Cancer After Castration	NCT00878436 Ferrari et al. (2019)
11	Vorinostat		2	Localized prostate cancer	NCT00589472
12	Pracinostat (SB939)		2	Recurrent or Metastasis Prostate Cancer	NCT01075308 Eigl et al. (2015)
13	Romidepsin		2	Prostate cancer	NCT00106418 Molife et al. (2010)
14	JBI 802 (LSD1/HDAC6 inhibitor)		2	Neuroendocrine prostate cancer (NEPC)	NCT05268666 Ojha et al. (2021)
15	Belinostat	Talazoparib	1	Metastatic Castration-resistant Prostate Cancer	NCT04703920 Ziadeh and Kourie, (2021)

Entinostat (SNDX-275, MS-275) is a class I and IV HDACi that is taken orally. *In vitro* and *in vivo*, entinostat decreases prostate cancer (PCa) growth and suppresses Treg cell function. In the small phase I investigation, Entinostat at the indicated dose levels in conjunction with the standard dose of enzalutamide demonstrated a promising safety profile (NCT03829930). Prostate cancer cells can develop in response to androgen. Enzalutamide, for example, may reduce the quantity of androgen produced by the body. Decitabine may inhibit tumor cell growth by inhibiting some of the enzymes required for cell proliferation. Decitabine with enzalutamide may be more effective in treating castration-resistant prostate cancer patients (NCT04471974).

Vorinostat inhibits class I and II HDACs. It has been demonstrated to reduce PC-3 xenograft tumors and decrease the growth of PC-3, DU-145, and LNCaP human prostate cancer cell lines. It causes Akt dephosphorylation by disrupting HDAC complexes bound to PP1, which results in more PP1–Akt association complexes and enhanced PP1-Akt association (Kulp et al., 2006). A phase II clinical trial (NCT00330161) was just completed on the medication (Bradley et al., 2009). Oral vorinostat is being tested in two other clinical trials at the same time. The Roswell Park Cancer Institute is conducting a phase I trial (NCT01174199) to compare vorinostat to intravenous temsirolimus in individuals with metastatic disease. The “Total Androgen-Receptor Gene Expression Targeted Therapy (TARGET) trial,” a National Cancer Institute phase II study (NCT00589472), evaluates neoadjuvant vorinostat with oral bicalutamide and intramuscular leuprolide acetate or subcutaneous goserelin acetate 4–8 weeks prior to radical prostatectomy. FT-7051 is an orally available, effective, and selective CBP/p300 inhibitor that has shown promise in preclinical models of prostate cancer, particularly those resistant to AR inhibitors like enzalutamide. The Courage Study (NCT04575766) is a multicenter, phase 1 open-label study evaluating the safety, pharmacokinetics (PK), preliminary anti-tumor activity, and pharmacodynamics (PD) of FT-7051 in men with metastatic castration-resistant prostate cancer (mCRPC) who have progressed amidst prior treatment and have been treated with at least one approved androgen receptor pathway inhibitor (NCT04575766).

In mCRPC patients whose disease has progressed on prior abiraterone or enzalutamide, a phase Ib open-label, dose-escalation study was conducted to assess the safety and efficacy of oral administration of GSK525762 in combination with either abiraterone plus prednisone (Arm A) or enzalutamide (Arm B) (Vaishampayan et al., 2018). In metastatic CRPC, a phase Ib/IIa clinical trial combining the BETi PLX2853 with abiraterone or olaparib has recently been begun (NCT04556617). Finally, a new phase II trial in men with CRPC (NCT04471974) combines enzalutamide (ZEN003694) and the immune checkpoint inhibitor pembrolizumab (NCT04471974). This trial comprises a group of patients with NEPC tumors. These findings show that BETi is a viable strategy worthy of further investigation. The possibility of combining BETi with immunotherapy or AR targeting medicines to treat advanced prostate cancer will be determined by the results of ongoing clinical trials.

Panobinostat (LBH589), an HDACi, resensitized CRPC models that had undergone EMT to ADT (Ruscetti et al., 2016). Despite these encouraging preclinical findings, clinical trials of single-agent HDACi have failed to show meaningful effectiveness (Eigl et al., 2015). Furthermore, a recent clinical trial with panobinostatin conjunction with the AR inhibitor bicalutamide revealed that the combination could be used to resensitize tumors to AR inhibition (Ferrari et al., 2019). In another phase Ib/IIa research, patients with metastatic CRPC who were resistant to enzalutamide and/or abiraterone were given enzalutamide and the BETi ZEN003694 (NCT02711956). In a subgroup of patients, the combination of enzalutamide and ZEN003694 was well tolerated and resulted in a prolonged PFS (Aggarwal et al., 2020).

Pracinostat, which has been designated as an orphan medication by the FDA for the treatment of AML, was also tested in a clinical phase 2 trial with 32 patients with CRPC. Even though just two patients had PSA reductions of more than 50%, the medicine (60 mg, 3 times per week, p. o.) was well tolerated, led to stable disease in 22% of patients, and reduced the amount of circulating tumor cells in nine others (Eigl et al., 2015). A phase 2 clinical trial with 35 CRPC patients tested the depsipeptide romidepsin. Two patients had a radiological partial response, 11 had stable disease, and 22 had progressive disease after receiving intravenous romidepsin (13 mg/m^2^). Despite the absence of grade 4 complications, 11 patients had to drop out of the study early, and romidepsin was not advised for additional phase 3 trials in CRPC (Molife et al., 2010). Vorinostat and romidepsin, on the other hand, caused drug-induced toxicities in a significant proportion of patients, forcing them to discontinue treatment. The relatively poor responses induced by the aforesaid first-generation HDACi when administered as a monotherapy in CRPC patients is the reason why no phase 3 studies in CRPC patients have been performed yet.

### Breast cancer

Breast cancer, a heterogeneous illness, is the most commonly diagnosed cancer in women globally and the second greatest cause of cancer-related death (Siegel et al., 2019). Breast cancer affects roughly 12% of American women during their lifetime, according to the American Cancer Society. Furthermore, approximately 2,300 men were diagnosed with breast cancer in 2015, with 440 dying as a result of the disease (DeSantis et al., 2015; Nyante et al., 2017). Males are not immune to breast cancer. Males usually have worse outcomes than females because to delays in diagnosis and estimates (Rizzolo et al., 2013; Kochan and Kovalchuk, 2015). The current classification of breast cancer is based on molecular subtypes, which reflect the tumor’s hormone response (Cava et al., 2015). Breast cancer can be divided into two primary classes and four groups based on particular molecular subtypes. Breast cancer can be divided into four intrinsic subtypes based on gene expression profiling: luminal A, luminal B (Luminal B1 and Luminal B2), HER2 loaded, and basal-like (Table 5). (Fragomeni et al., 2018). Estrogen receptor (ER), progesterone receptor (PR), or the human epidermal growth factor receptor2 (HER2/ERBB2) protooncogenic receptor are expressed in around 90% of breast cancer patients. In the luminal A subtype, mutations in GATA3, PIK3CA, and MAP3K1 were frequently found (Koboldt et al., 2012). The Luminal B2 subtype of HER2-positive breast cancer, which overexpressed GATA3, BCL2, and ESR1 genes, was found to account for roughly half of all HER2-positive breast cancer subtypes (Wu et al., 2015). The frequency of p53 mutations was higher in luminal B1 than in luminal A, although the prevalence of PIK3CA mutations was lower.

**TABLE 5:** Classification of breast cancer.

Category	Hormone receptor status	Category	HER2 status
Luminal	ER,PR positive	Luminal A	HER2 negative
		Luminal B	HER2 positive
Non-luminal	ER,PR negative	HER2+	HER2 positive
		Triple negative	HER2 negative

The overexpressed genes in the luminal subtype were down-regulated or deleted in the HER2 positive subtype. There is no clinically-proven type-specific therapeutic target for the 10% of breast cancer cases that are negative for ER, PR, and HER2, and hence are designated “triplenegative,” and only genotoxic chemotherapy is utilised (Sørlie et al., 2001). TNBC is a more aggressive subtype of breast cancer that, regrettably, remains a clinical challenge to treat due to its poor prognosis, aggressiveness, and lack of targeted medicines. TNBC is divided into six molecular subtypes: two basal like classes (BL1 and BL2), immunomodulatory (IM), mesenchymal (M), mesenchymal stem cell (MSL), and luminal androgen receptor (LAR) (Eckstein, 2011). Anti-estrogens (e.g., aromatase inhibitors, tamoxifen, fulvestrant) and HER2-targeted medicines have greatly increased their survival (Giuliano et al., 2011; Rexer and Arteaga, 2012). However, some tumors grow *de novo* or gain resistance to anti-estrogen and HER2-targeted medicines despite these treatments, and these tumors can recur (Tryfonidis et al., 2016). In order to treat breast cancer, new therapeutic targets must be developed, given the disease’s high prevalence and severity.

### Development HDACi against breast carcinoma

**FIGURE 2:**
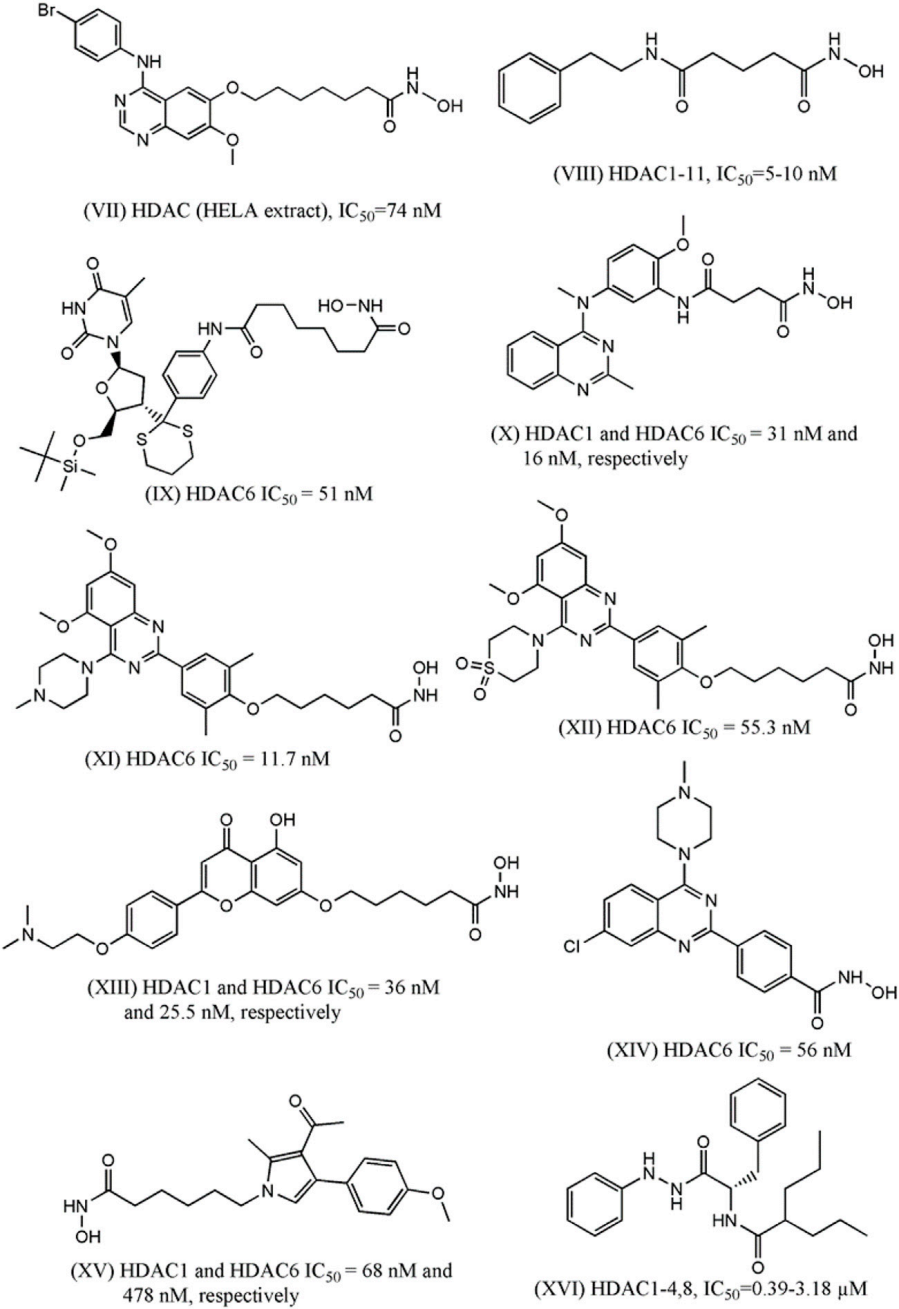
Structures of HDACi designed in recent years that depicted anti-proliferative potential against the breast carcinoma cell lines.

In 2016, Peng et al. designed and synthesized eighteen N-phenylquinazolin-4- amine hybrids as dual inhibitors of vascular endothelial growth factor receptor 2 (VEGFR-2) and HDACs considering the roles of these receptors in cancer progression (Peng et al., 2016). Upon interacting with its endogenous substrate VEGF, the tyrosine kinase receptor VEGFR-2 initiates downstream signaling leading to tumor angiogenesis, proliferation and migration. Being a promising target of cancer therapy, several agents have been developed as VEGFR-2 though frequent emergence of drug resistance that restricted their therapeutic potential. Therefore, the development of multitarget inhibitors with activity against both VEGFR-2 and HDACs may be promising strategy. The designing strategy adopted by the investigators was simple and interesting as they attempted to combine the aromatic moiety of vandetanib (a VEGFR-2 inhibitor) with the side chain of HDACi vorinostat. Among 18 synthesized derivatives, one compound (VII, Figure 2) depicted IC_50_ values of 2.2 nM and 74 nM against HDACs (HELA cell nuclear extract containing the mixtures of HDACs) and VEGFR-2, respectively. At the same time the cell proliferation assay revealed that this compound is active against breast carcinoma cell line MCF-7 with IC_50_ of 0.85 µM. When this compound was tested against different HDAC isoforms such as HDAC-1, HDAC-2, HDAC-6 and HDAC-8, maximum potency was observed against HDAC-1 (i.e., IC_50_ = 1.8 nM) followed by HDAC-2 (i.e., IC_50_: 3.3 nM), HDAC-8 (i.e., IC_50_: 4.6 nM) and HDAC-6 (i.e., IC_50:_ 16.4 nM). In the same year, Gromek and co-workers (Gromek et al., 2016) attempted to develop potent HDACi with a hybrid structure (SCA-vorinostat, Shown in Figure 2, VIII) formed with vorinostat and santacruzamate A (SCA), a natural HDACi (previously identified by the investigators) with micromolar potencies against various isoforms of HDACs. Similar to SCA, SCA-vorinostat was found to be active against multiple HDAC isoforms and forty derivatives of these compounds were prepared. When these hybrids compounds were tested against breast carcinoma cells, two silylated derivatives were found to have promising antiproliferative activity against MCF-7 cell line and interestingly the potency (GI_50_ of 13.3 and 23.7 nM) of these compounds against this breast carcinoma cell line was even higher than that of vorinostat (i.e., GI_50_ 29.1 nM). Interestingly, these compounds were in fact tested against a panel of cell lines that also included triple negative breast carcinoma cell MDA-MB-231, colon carcinoma cell HCT-116, lymphoma cells (Hut-78 and Molt-4) as well as against non-cancerous peripheral blood mononuclear cell PBMC. However, no significant potency was noted against any of these cell lines including MDA-MB-231 indicating that these compounds may selectively inhibit normal breast carcinoma cells following unique mechanisms. In 2016, Saha et al. (2016) reported one investigation where twelve compounds containing triazole and hydroxyacetamide moieties that were tested against HDACs and MCF-7 cells. The most potent HDACi in this series depicted IC_50_ of 90 nM which is close to the potency of vorinostat (IC_50_: 56 nM) but the HDAC inhibitory activity did not match well with antiproliferative activity of these derivatives against MCF-7 that range from growth inhibitory (GI_50_) values of 20–60 µM. In 2018, one research investigation was reported by Yamashita et al. (2018) describing the design of dual inhibitors of HDACs and DNA topoisomerase II. The latter enzyme is a well-known target for inhibition of cancer progression due to its role in the regulation of DNA topology. Some hybrid compounds were prepared by combining the side chain of HDACi Trichostatin and a lactone compound that was proved to be a potent inhibitor of DNA topoisomerase II. One of these hybrids was found to be dual inhibitors of both enzymes and further modification of this structures led to six of its derivatives that were found to be potent inhibitors of DNA topoisomerase II as well as HDACs and at the same time, these were also found to be antiproliferative against breast (MCF-7), colon (HCT-116) and prostate (DU-145) cancer cells. For example, the most potent compound of this series (IX, Figure 1) had growth inhibitory (GI_50_) values of 3.24 µM, 3.39 µM and 3.98 µM against MCF-7, HCT-116 and DU-145. Another important investigation was reported in the same year (Chen et al., 2019), where a series of methylquinazoline derivatives were rationally designed based on previously conducted in silico fragment-based analyses that had projected this fragment as a potential surface recognition cap group. The synthesized compounds were first tested for their antiproliferative activity against colon cancer cell line HCT116 and based on the results, eleven compounds were picked for enzymatic assays against HDAC1 and HDAC6. Significantly all these compounds depicted nanomolar potency against these enzyme isoforms and more importantly, these compounds also had sufficient selectivity towards these two specific HDAC isoforms. A series of solid and hematologic tumor cell lines were then chosen to estimate the overall antiproliferative potential of some of these inhibitors and two compounds depicted IC_50_ values of 2.65 and 7.41 nM against MCF-7 cell line. Based on MCF-7/ADR xenograft model, one compound (X, Figure 2) was finally projected as a potential lead molecule for cancer therapy. Focusing on the treatment of triple negative breast carcinoma, Yao et al. (2020) later selected the same quinazoline moiety to synthesize more than forty derivatives and each of these was tested against HDAC-6 by enzymatic assay and MDA-MB-231 by cell viability assay. Two compounds with high inhibitory potential against both these targets were then selected for pharmacological assays where these were proved to promote autophagy, apoptosis while suppressing migration of this breast carcinoma cells. More significantly, improved pharmacokinetic profiles were noted with these derivatives as compared to vorinostat. Two of the most potent compounds (XI and XII) in this series are shown in Figure 2.

More recently, Wei et al. (2020) designed some flavone- and isoflavone-based HDACi considering pharmacological versatility of these moieties. Choosing these moieties as hydrophobic capping groups, more than 25 derivatives were synthesized and tested for inhibitory potential against HDACs. The most potent one (XIII, Figure 2) was then tested for isoform selectivity that led to the observation that its more selective towards HDAC-1,2,3, and 6. A number of biological assays were then conducted to establish strong antiproliferative activity of this lead molecule against multiple TNBC cells (i.e., MDA-MB-231, MDA-MB-468, BT-549, and Sum-159), HER-2 negative breast carcinoma cell (i.e., MCF-7, T47D), breast carcinoma cell (i.e., BCAP-37), pancreatic carcinoma (i.e., PANC-1, PC-3), melanoma, hepatocarcinoma, lung carcinoma as well as normal human cell lines. The same compound, with improved pharmacokinetic profiles, was found to downregulate HDAC-induced STAT3 *in vivo* in some breast cancer cells to enhance anti-tumor activity.

In another investigation (García et al., 2020), fluorescent coumarin-hydroxamic acid derivatives were designed as HDACi. The designed compounds, which were actually hybrids of vorinostat and coumarin, were initially tested against two breast carcinoma (i.e., BT-474 and MDA-MB-231) and one prostate cancer (i.e., PC-3) cell lines to choose the most potential hits and some of these were found to downregulate the expressions of cell-cycle regulatory genes such as p21, p53 and cyclin D1 (CD1) in both breast and prostate cancer cells. However, enzyme inhibition activity against any HDAC of these derivatives were not reported though molecular docking analyses hinted that some of these compounds may bind to the catalytic sites of class I HDACs.

The role of quinazoline moiety was further investigated by Yao et al. (2021) with a purpose to design dual mTOR/HDACi. The mTOR is one of the key members in pI3K-AKT-mTOR pathway that have been implicated in the progress of several carcinomas. The investigators correctly identified that quinazoline moiety, which is present in the very potent mTOR inhibitor KU-0063794, may be explored to develop hybrid structures with activity against mTOR and HDACs. A total 28 derivatives were synthesized and tested against mTOR and HDAC6 but maximum inhibitory potential was obtained with a compound (shown in Figure 2 as XIV) that depicted IC_50_ of 56 nM against HDACs and IC_50_ of 133.7 nM against mTOR. This compound depicted promising anti-proliferative activity against TNBC cell like MDA-MB-231 and at the same time also promoted autophagy and apoptosis in this cell in a dose-dependent manner. The anti-migratory action was also noted in the cells treated with this compound. Very recently Singh and co-workers (Singh et al., 2021) attempted to develop some pyrrole based HDACi keeping the hydroxylamine moiety intact as zinc binder domain. After synthesizing a series of 4-substituted-methyl 6-(3-acetyl-2-methyl-1H-pyrrol-1-yl)-hexanoate and 4-substituted-6-(3-acetyl-2-methyl-1H-pyrrol-1-yl)-N-hydroxyhexanamide derivatives, some most potent derivatives were tested against breast cancer cell line MCF-7 along with leukemia, lung and cervical cancer cell lines. One compound (XV, Figure 2) depicted total growth inhibition (TGI) at 53.7 µg/ml and this compound also showed IC_50_ of 68 nM and 478 nM against HDAC-1 and HDAC-6, respectively. While designing novel HDACi, hydroxamic acid remained the most frequent choice as zinc binding group even though this group may be replaced with other zinc binding groups as well. Recently, Ibrahim et al. (2020) designed structurally diverse HDACi that consisted of valproic acid as the cap group whereas these designed compounds contained a range of zinc binding groups (e.g., carboxy, hydrazine, etc) as zinc binding domain. These valproic acid conjugates were tested for their antiproliferative activity with multiple human solid cancer cell lines including breast cancer cell MCF-7. Based on the cytotoxicity results, a few compounds were then assayed against nine different HDAC isoforms (i.e., HDAC-1-9) to assure their potency and selectivity towards class I HDAC enzyme isoforms. One compound (XVI, Figure 2) was then selected for cell cycle analyses conducted with MCF-7 cells to infer that it is likely to lead towards Pre-G1 apoptosis and cell growth arrest at G2/M. Furthermore, the investigators also resorted to *in vivo* assay to confirm that this novel compound is capable of reducing the size of tumor as well as number of tumor cells in mice model. In recent reports, Bingul et al. (2021) helped identifying some indole derivatives to enhance the cytotoxic efficiency of vorinostat against breast cancer cells such as MCF-7 and MDA-MB-231.

### Histone deacetylases and breast cancer

At the estrogen- and progesterone-mediated signaling pathways, HDACs play a crucial role in transcriptional control. Acetylation has been discovered to be a crucial mediator at numerous locations along this pathway, affecting both ER transcription and turnover (Thomas and Munster, 2009). The development of breast cancer has been linked to a disruption in the equilibrium between HATs and HDACs. Zhang et al. (2004) discovered that hormone-positive breast cancer patients with small tumors and low histological grade had increased expression of HDAC6 mRNA, indicating that they were more receptive to endocrine treatment and had a better prognosis. Krusche proposed HDAC 1 as an independent breast cancer prognostic marker in 2005. HDAC 1 expression profiling could be relevant in the clinic to help patients with breast cancer receive individualized, risk-directed adjuvant systemic therapy (Krusche et al., 2005). HDAC4 upregulation was discovered in breast cancer cells in 2006, compared to lung and colon cancer cells (Özdağ et al., 2006). Lee et al. (2008) approved the potential function of HDAC6 in anchorage independent breast cancer cell proliferation. Ververis and Karagiannis (2012) found higher expression of Class I HDACs in breast cancer tissue than Class II enzymes. HDACs inhibits the expression of GABARAPL1, an autophagy-related gene, and promotes breast cancer development (Hervouet et al., 2015). Furthermore, tumor differentiation and tumor cell proliferation are linked to the expression levels of the histone-modifying enzymes HDAC2, LSD1, and SIRT1 (Derr et al., 2014). The global reduction of monoacetylated lysine 16 of histone H4 (H4K16) is a common occurrence in cancer, and low levels of H4K16 acetylation have been indicated as an early event in breast cancer (Falahi et al., 2014). Suziki et al. discovered that ductal carcinoma *in situ* and invasive ductal carcinoma have lower levels of ac-H4 and ac-H4K12 acetylation than normal breast epithelium (Karsli-Ceppioglu et al., 2014). H3K4ac has been linked to both early and late phenotypes of breast cancer cells. H3K4ac enrichment is seen at promoters of genes linked to cancer-related phenotypic features like oestrogen response and epithelial-to-mesenchymal transition pathways (Messier et al., 2016). As a result, HDACs are critical for breast cancer pathogenesis and progression, giving new therapy options for the disease.

### Preclinical studies of HDACi in breast cancer

HDACi have been tested in all breast cancer subtypes because preclinical research shows that this class of drug can target breast cancer in a variety of ways, including relief of transcriptional repression with an impact on the epithelial-mesenchymal transition (EMT), reactivation of silenced oestrogen receptor (ER) in hormone receptor-negative tumors, restoring the sensitivity of hormonal therapy in estrogen-positive tumors, and modulation of transcriptional repression with an impact on the epi (Connolly et al., 2017). It was previously reported that HDACi regulate 8%–20% of genes at the transcriptional level by inhibiting HDACs function on histone tails, and that they could also target gene transcription *via* an indirect mechanism by inhibiting HDACs interactions with non-histone proteins, as HDACs act on a variety of proteins other than histones, including transcription factors, enzymes, and HDACs themselves (Glaser et al., 2003). HDACi inhibits HDACs activity, resulting in hyper-acetylation of histone lysine residues. HDACs plays a critical role in breast cancer treatment (Contreras-Leal et al., 2016). HDACs block HDAC’s catalytic activity by chelating the zinc co-enzyme factor. HDACi can inhibit breast cancer in a variety of ways, and they can also help with the therapy of breast cancer. HDACi has the potential to disrupt cell mitosis. HDACi CG-1521 inhibits the production of mitotic spindles and prevents abscission during cytokinesis, which leads to death in inflammatory breast cancer cells (Chatterjee et al., 2013). HDACi are also known to induce apoptosis by regulating anti- and/or pro-apoptotic molecules such as the Bcl-2 family of molecules (Johnstone and Licht, 2003), and the mechanism underlying the suppression of Bcl-XL protein by HDACi has been reported to be regulated at the transcriptional or translational level in several carcinomas, including TNBC (Johnstone and Licht, 2003). Although HDACi appear to be potential anti-cancer medications in theory, their usage as monotherapy for solid tumors is still limited, and most trials examining them combine them with another anti-cancer drug (Wang et al., 2016). Preclinical evidence suggests that HDACi, as a single drug candidate or in combination with other anticancer medicines, have a wide range of anticancer effects in several cancer cell lines and cancer xenograft models by targeting many cancer pathways.

Vorinostat, TSA, belinostat, panobinostat, givinostat, resminostat, abexinostat, and quisinostat are examples of pan-HDACi, while ricolinostat, pracinostat, and CHR-3996 are instances of selective hydroxamates. Vorinostat, TSA, panobinostat, and belinostat have all been studied extensively in different BC cell models. The FDA has approved vorinostat and panobinostat for the treatment of cutaneous T-cell lymphoma and multiple myeloma, respectively (Andreu Vieyra and Berenson, 2014). Three HDACi, vorinostat, and panobinostat, have been approved by the FDA and are currently being tested in clinical trials for breast cancer patients as a single agent or in combination with other standard therapies such as chemotherapies, aromatase inhibitors (exemestane), or SERM (tamoxifen) (Munster et al., 2011). Vorinostat is a hydroxamic acid-based HDACi that has been proven to block the majority of HDACs (class I, II, and IV), making it a pan inhibitor. Vorinostat inhibited the proliferation of TAMR/MCF-7 BC cells (tamoxifen-resistant) *via* inducing apoptosis and autophagic cell death, according to Lee et al. In mice with TAMR/MCF-7 tumors, vorinostat also inhibited tumor cell proliferation (Lee et al., 2012). In a mouse model of triple-negative BC, Palmieri et al. (2009) found that vorinostat can generate dsDNA breaks and can prevent brain metastasis (62% large metastases compared to untreated groups). Vorinostat can activate heat shock protein (hsp)90 hyperacetylation, diminish hsp90 binding to ERα, increase polyubiquitylation, and decrease ERα expression in ERα-positive BC cells, according to Fiskus et al. (Fiskus et al., 2007). Vorinostat was also discovered to limit the capacity of 4T1 BC cells to migrate and invade. Vorinostat inhibited 4T1-luc cell metastasis *in vivo*, according to the results of the same study’s *in vivo* trials (Chiu et al., 2013). Terranova-Barberio et al. found that vorinostat therapy resulted in an 18-fold increase in PD-L1 and HLA-DR expression in triple-negative BC cells (Terranova-Barberio et al., 2017).

TSA is an HDACi that comes from nature (Damaskos et al., 2017). TSA was discovered to make hormone-receptor-negative BC cells receptive to tamoxifen by altering the transcriptional activity of ER in ER-negative BC cells (Jang et al., 2004). Chen et al. (2017) have revealed that co-treatment of TSA with BEZ235 (a PI3K/mTOR/AKT pathway inhibitor) can cause apoptosis and mediate strong anticancer effects in MCF-7, T47D, and MDA-MB-231 BC cells. The same study also found that co-treatment of TSA with BEZ235 inhibited the growth of MDA-MB-231 tumors in mouse xenograft models. TSA and CG-1521 (hydroxamate-based HDACi) can induce apoptosis and cause cell cycle arrest in SUM149PT and SUM190PT inflammatory BC cell lines, according to Chatterjee et al. (2013). Panobinostat can greatly increase histone acetylation, cell cycle arrest, and trigger apoptosis in BC cells, as per preclinical studies. Tate et al. (2012) found that panobinostat can reduce proliferation and induce histone acetylation in triple-negative BC cell lines MDA MB157, MDA-MB-231, MDA-MB-468, and BT-549. Panobinostat can also re-express the silenced ER gene in triple-negative BC cells *via* restoring heterochromatin-associated proteins without promoter hypermethylation, according to Zhou et al. (2007). Furthermore, a study found that treating hormone-responsive BC cells with panobinostat and letrozole together suppressed aromatase expression synergistically, implying that panobinostat and letrozole combined therapy is an appropriate way to target hormone receptor-positive/aromatase-positive BC cells (Chen et al., 2010).

Another FDA-approved medication for the treatment of PTCL is belinostat. Hsu et al. recently found that belinostat inhibited the proliferation of MDA-MB-231, SKBR-3, and MCF-7 BC cell lines and triggered apoptosis in a caspase-dependent manner (Hsu et al., 2018). Belinostat or vorinostat were also reported to reduce the proliferation of a triple-negative BC cell line panel (8 cell lines) and tumor growth in triple-negative BC xenografts when used in combination with olaparib (an FDA-approved anticancer medication) (Marijon et al., 2018). In MCF-7 BC cells treated with belinostat, Androutsopoulos and Spandidos observed reduction of cell growth, strong HDACs inhibition, and elevation of acetylated tubulin levels (Androutsopoulos and Spandidos 2017). Valproic acid (VPA) is a valeric acid-derived short-chain fatty acid (Damaskos et al., 2017). Tian et al. (2017) recently discovered that VPA and hydroxyurea (a ribonucleotide reductase inhibitor) can limit the proliferation of MCF-7 BC cells synergistically by inhibiting RPA2 and increasing the Rad51-mediated homologous recombination DNA repair pathway. By suppressing the activity of HDAC1 and modifying the methylation state of H19 by induction of the enzyme DNA methyltransferase 1 (DNMT1) expression, Hao et al. (2017) found that VPA may induce death in A549 BC cells and reduce the production of the H19 oncogene.

The sodium salt of butyric acid is designated as NAB (Smith et al., 1998). NAB can trigger apoptosis in MCF-7 and MDA-MB-468 BC cell lines *via* oxidative stress, according to a recent study (Salimi et al., 2017). The similar effects of NAB have been demonstrated in MCF-7 BC cells by Louis et al. (Louis et al., 2004). Furthermore, *via* regulating Fas signaling, NAB has been shown to cause apoptosis in MCF-BC cells in a p53-independent way (Chopin et al., 2002). Entinostat is a class I HDACi that is synthesised (Knipstein and Gore, 2011). Schech et al. (2015) found that entinostat significantly reduced TICs from triple-negative BC cells in a study. Furthermore, entinostat treatment decreased the number of CD44 (high)/CD24 (low) cancer stem cells, aldehyde dehydrogenase 1 (ALDH1) levels, and TIC marker expression (Oct-4, Bmi-1 and Nanog). According to Shah et al. (2014), entinostat can reverse epithelial-mesenchymal transition (EMT) in triple-negative BC cells by decreasing the binding of transcription factors Snail and Twist to the E-cadherin promoter. Entinostat was discovered to sensitise HER2-positive BC cells to trastuzumab/lapatinib treatment, supporting the use of entinostat/lapatinib and trastuzumab in HER2-positive BC patients.

Cyclic tetrapeptides are natural chemicals found in fungi and bacteria from the sea (Li and Seto, 2016). Romidepsin is a selective class I HDACi that has been approved by the FDA for the treatment of CTCL patients. Primary and metastatic cancers were effectively suppressed by a combination of romidepsin and paclitaxel treatment. In another study, romidepsin and oncogenic H-Ras stimulated the ERK pathway in H-Ras-transfected MCF10A mammary cells, resulting in the activation of Nox-1 and reactive oxygen species (ROS) and apoptosis (Choudhary et al., 2010). Several cancer cell models have been used to test the preclinical efficacy of sirtuin inhibitors such sirtinol, cambinol, EX527, and suramin. By decreasing the expression of SIRT1/2 in MCF-7 BC cells, Wang et al. found that sirtinol can trigger apoptotic and autophagic cell death (Wang et al., 2012). In sirtinol-treated MCF-7 BC cells, researchers were able to induce a senescence-like growth arrest by inhibiting the Ras/MAPK signaling pathway (Ota et al., 2006). Sirtuins are located in a variety of subcellular sites in mammals, including the nucleus (SIRT1, 2, 6, and 7), mitochondria (SIRT3, 4 and 5), and cytoplasm (SIRT1 and 2), and are thought to play a role in cell survival, metabolism, ageing, and genetic integrity (Haigis and Sinclair, 2010).

### Registered HDACi in clinical trials of breast cancer

HDACi have failed to exhibit highly effective anticancer efficacy in breast cancer clinical trials as single medicines. HDACi, on the other hand, have become a popular component of breast cancer treatment regimens (Trapani et al., 2017). Hormone therapy resistance is a problem in treating estrogen receptor (ER) positive breast tumors, hence researchers have looked into using HDACi in combination with hormone therapy. The first clinical trial combining vorinostat and tamoxifen for hormone therapy-resistant breast cancer was completed by researchers (NCT00365599) (Munster et al., 2011). This study looked at restoring hormone sensitivity to tamoxifen in advanced breast cancer patients who had progressed on prior hormone therapy. The 43 individuals who participated in this study had their H4 acetylation and HDAC2 expression induced by vorinostat in their peripheral blood mononuclear cells. The addition of the HDACi vorinostat to tamoxifen hormone receptor-positive breast tumors resulted in tumor regression or prolonged disease stability in 40% of patients who had progressed on earlier hormonal therapy and chemotherapy, as per the findings.

Entinostat decreased MDSCs and the regulation of MDSC CD40 expression in breast cancer patients, while also increasing HLA-DR expression on CD14^+^ monocytes. These findings support the use of entinostat in conjunction with immune checkpoint inhibitors as a treatment option (Tomita et al., 2016). In addition, a phase III trial in hormone receptor-positive breast cancer patients will soon begin assessing endocrine therapy with the HDACi, entinostat, or placebo (NCT02115282). Vorinostat was studied as a single drug in early BC clinical investigations. However, HDACi’s overall clinical effectiveness as single treatments in solid tumors has not always been favourable. Rubin et al. (2006) conducted a Phase I research in patients with advanced cancer, including BC, to assess the safety, tolerability, and pharmacokinetics of single and multiple doses of vorinostat, as well as the effects of a high-fat diet on vorinostat pharmacokinetics. The results of research showed that one (stage IV BC) of four BC patients who took vorinostat [400 mg on days 1–20 (fasted) and 5 (fed)] and were fed a high fat diet for >15 months maintained stable disease (SD).

In addition, patients with advanced HR-positive, HER2-negative breast cancer whose disease progressed after nonsteroidal aromatase inhibitors were used were enrolled in a multicenter, randomised, double-blind, placebo-controlled phase III research (E2112). This phase III clinical trial was based on the results of a prior ENCORE301 phase II investigation, which found that combining entinostat and exemestane therapy improved PFS and OS (Yardley et al., 2013). Patients were given either oral 25 mg exemestane once daily and 5 mg entinostat or placebo 5 mg once weekly in this trial. According to the findings, the median PFS of exemestane plus entinostat was 3.3 months and 3.1 months, respectively, with no significant difference.

In metastatic advanced triple-negative breast cancer (TNBC) patients, a phase II single-arm clinical trial was done to examine the efficacy and safety of a combination treatment of tucidinostat/chidamide with cisplatin. Tucidinostat/chidamide and cisplatin, 20 mg twice weekly for 2 weeks and 75 mg/m2 on a 21-days cycle, were given to women with TNBC (Meng et al., 2021). Table 6 lists some of the most recent clinical trials for breast cancer treatment.

**TABLE 6:** HDACi in clinical trials of breast cancer.

Sl no.	Drug	Combined target	Phase	Cancer type	References
01	Tucidinostat	Exemestane-steroidal aromatase inhibitor, hormonal therapies	Phase III	Hormone receptor-positive (HR+) and HER2 negative breast cancer	Jiang et al. (2019)
02	Entinostat	Exemestane-steroidal aromatase inhibitor, hormonal therapy	phase III	Hormone receptor-positive (HR+) and HER2-negative breast cancer	Connolly et al. (2021)
03	Tucidinostat	Cisplatin, chemotherapy	Phase II	Triple-negative breast cancer	Meng et al. (2021)
04	Romidepsin	Cisplatin and nivalumab	Phase I/II	Triple-negative breast cancer	NCT02393794 Wang X. et al. (2019)
05	Entinostat	Exemestane	Phase III	Hormonereceptor-positive BC	NCT02115282 Yardley et al. (2013)
06	Entinostat	Atezolizumab	Phase I/II	Triple-negative breast cancer	NCT02708680 Page et al. (2019)
07	Entinostat	Nivolumab and ipilimumab	Phase I	Advanced HER2- negative BC	NCT02453620 National Cancer Institute (2022)
08	Vorinostat	Tamoxifen and pembrolizumab	Phase II	ER-positive BC	NCT02395627 (Munster, 2020)
09	Entinostat	Capecitabine	Phase I	BC following neoadjuvant chemotherapy	NCT03473639 Millard et al. (2019)
10	Belinostat	Ribociclib	Phase I	Metastatic breast cancer	NCT04315233 Wawruszak et al. (2021b)
11	Vorinostat	Olaparib	Phase I	Metastatic breast cancer	NCT03742245 Wawruszak et al. (2021a)
12	Chidamide		Phase II	Breast cancer	NCT05400993 Cogliati et al. (2022)
13	Tucidinostat	Capecitabine and endocrine therapy	Phase II	Breast cancer	NCT05411380 Wang, (2022)
14	Tinostamustine (EDO-S101)		Phase I and II	Triple-negative breast cancer	NCT03345485 Mita et al. (2019)
15	Belinostat	Talazoparib	Phase I	Metastatic breast cancer	NCT04703920 Pham et al. (2021)
16	Entinostat	Capecitabine	Phase I	Metastatic breast cancer	NCT03473639 Millard et al. (2019)
17	Vorinostat	Pembrolizumab and tamoxifen	Phase II	Stage IV breast cancer	NCT04190056 Schröder et al. (2021)

### Ovarian cancer

Most ovarian cancers are either ovarian epithelial cancers (cancer that begins in the cells on the surface of the ovary) or malignant germ cell tumors (cancer that begins in egg cells). With a survival rate of 47%, malignant ovarian cancer has the highest mortality rate compared to other cancers of gynecological origin (Moufarrij et al., 2019). The most characteristic trait of which is a papillary serous in its histology. The ovarian cancers are thus subdivided into high grade and low grade serous ovarian cancers. The incidence of about 14%–20% of all ovarian cancers are attributed to hereditary predisposition, mutations of specific genes (BRCA 1 & 2) and the loss of their function or genes encoding proteins that complex with BRCA proteins, such as BRIP1, RAD51C, RAD51D, and FANCM, which are responsible for mechanisms of DNA repair, along with the loss of mismatch repair function is what causes the genetic instability. This instability along with mutations in the tumor suppressor gene (Tumor protein-TP53) leads to high grade EOC (Moufarrij et al., 2019).

The major issue in treating ovarian cancer is its late diagnosis, causing the treatment to begin in advanced stages (stage III or IV). This is due to absence of distinct symptoms specific to early stage EOC and the lack of biomarkers for screening of EOC (Yang et al., 2018). The usual treatment protocol consists of surgery and cytoreduction for reduction of tumor volume followed by chemotherapy with a platinum agent and a taxane. Even though the initial response shows great promise, resistance to chemotherapy poses problems in treating ovarian cancers, caused by recurrent tumors. Many other targeted therapies have been used, namely PARP inhibitors, which targets angiogenesis, however the response was unsatisfactory (Yang et al., 2018). The heterogeneous nature of ovarian cancer makes selection of the right drug very difficult, suggesting that a multi-targeted treatment approach would be more effective in treating ovarian cancer. The limitations of the aforementioned mentioned therapies led the way for the use of novel epigenetic therapies to treat ovarian cancer (Khabele, 2014).

### Development HDACi against ovarian carcinoma

Only a few investigations were reported in recent years where ovarian carcinoma cells were tested for HDACi. In 2017, an investigation (Stenzel et al., 2017) was reported by Stenzel and co-workers (2017) that involved syntheses of aloxyurea-based HDACi to improve the potency of cisplatin in various chemo-resistant cancer cells. These aloxyurea derivatives, which contained structural similarity with ricolinostat, depicted selective inhibition of HDAC-1 and HDAC-6 over other HDACs and also displayed satisfactory inhibitory potential against ovarian cancer cells A2780 and A2780CisR. Further studies conducted with chemo-resistant cancer cells (i.e., A2780CisR and Cal27CisR) revealed that some of these potent derivatives actually increase the potency of cisplatin in a synergistic manner. Further, Andrade et al. (2018) explored the moiety of natural HDACi santacruzamate A to produce seven synthetic compounds that were tested against a panel of human cancer lines that included breast cancer cells MCF-7 and MDA-MB-231 as well as ovarian cancer cell TOV-21G to have moderate micromolar potencies. Even though *in vitro* potencies of these compounds against HDACs were not reported, further investigation revealed that the most potent compound of this series may indeed enhance DNA damage and may promote apoptotic cell death through intrinsic pathway.

### HDACs and ovarian cancer

**FIGURE 3:**
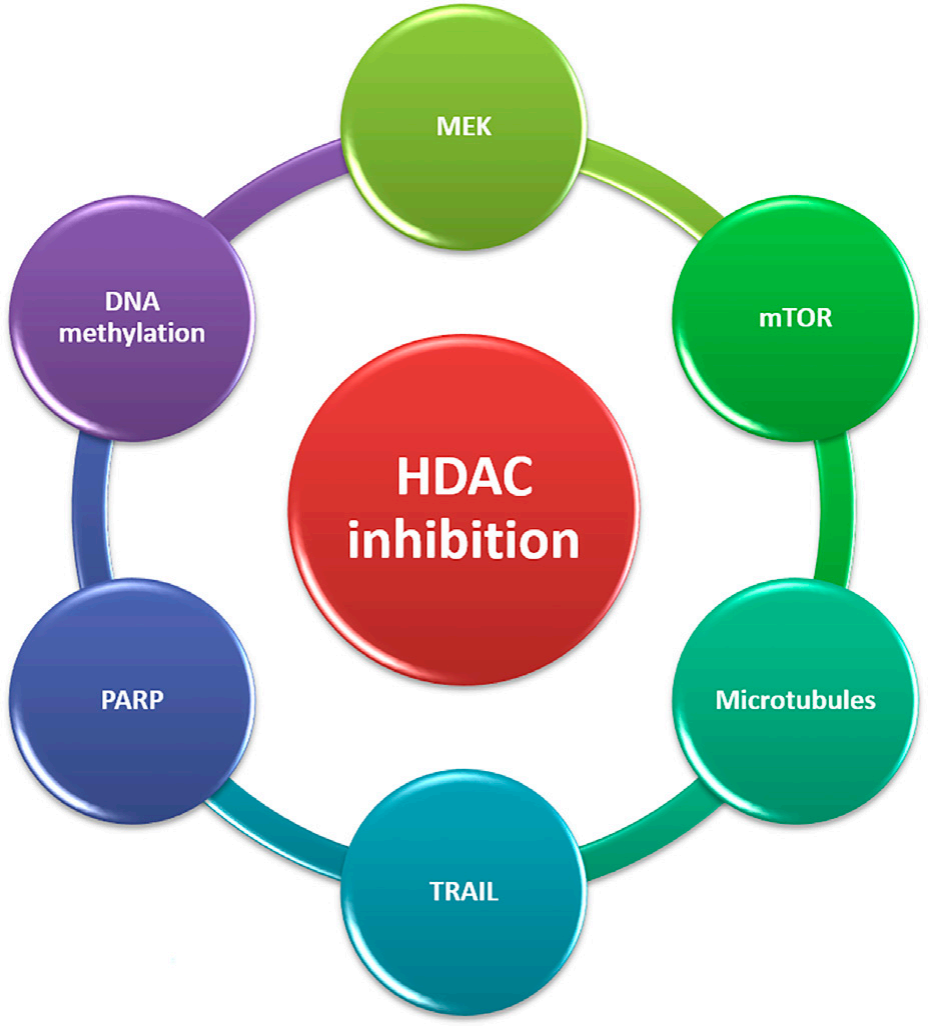
Targets for combination therapies with HDACi in CRPC. CRPC, castration-resistant prostate cancer; HDACi, Histone deacetylase inhibitors; TRAIL, TNF-related apoptosis-inducing ligand; MEK, MAPK/ERK kinase.

**FIGURE 4:**
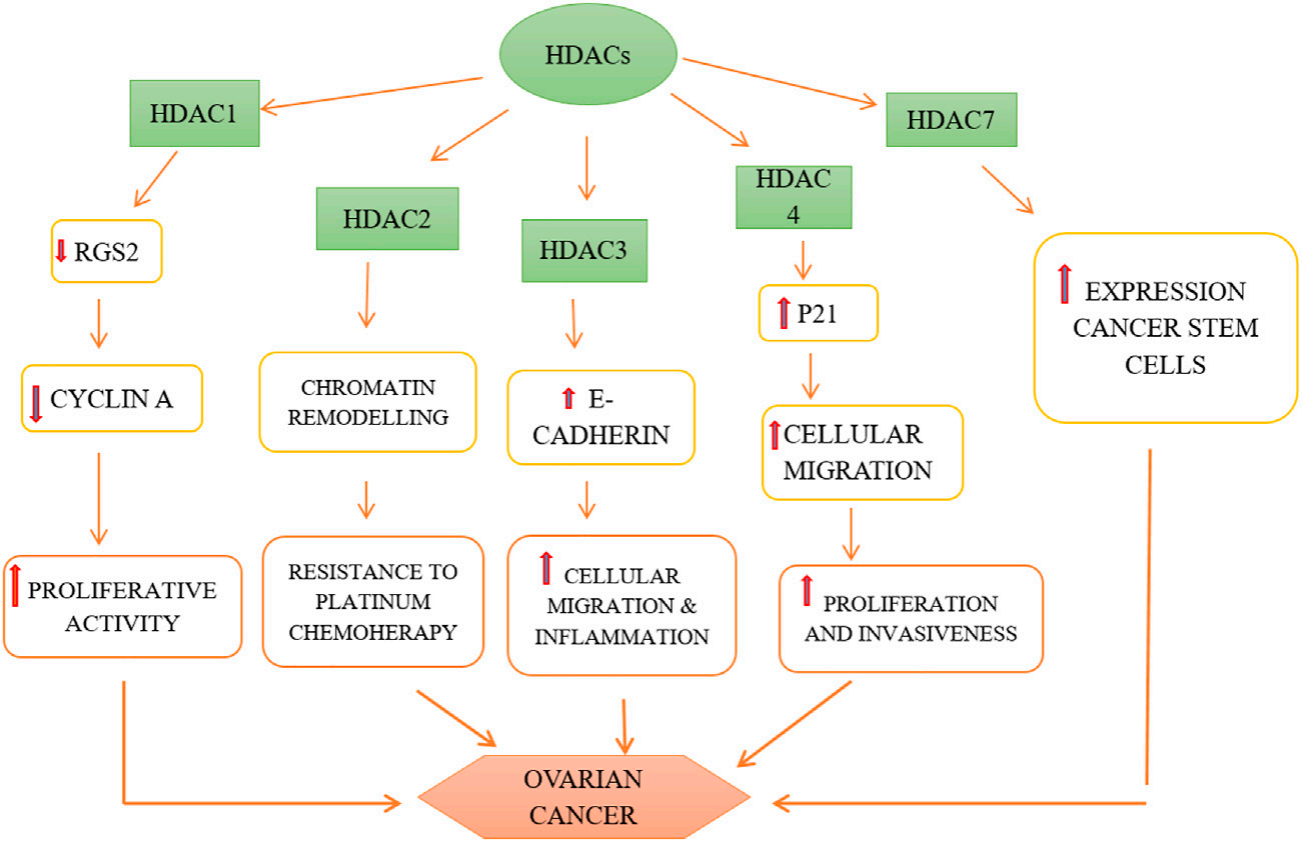
Role of HDACs in pathogenesis of ovarian cancer.

HDACs 1, 2, and 3 i.e., belonging to class I are over-expressed in ovarian cancer cells, namely high grade serous, mucinous, endometrioid as well as clear cell type of EOC and promote carcinogenesis (Yano et al., 2018). The role of HDACs in ovarian cancer is depicted in Figure 3. It has been observed that HDACs belonging to class I are over-expressed in tumor cells with high proliferative activity and this is also the underlying cause of poor prognosis of malignant ovarian tumor (Eckschlager et al., 2017). Regulator of G-protein Signaling 2 (RGS2) inhibits G-protein coupled receptors (GPCRs) by causing the deactivation of heterotrimeric G-proteins. It has been observed that RGS2 levels dropped sharply in chemo-resistant ovarian epithelial cells as compared to chemo-sensitive cells. HDAC1 are shown to down-regulate this RGS2 along with the promotion of cyclin A, thereby enhancing cellular proliferation (Smith et al., 2017). Resistance to platinum-based chemical therapies in EOCs is achieved by chromatin remodeling *via* HDAC2 (Yang et al., 2018). Suppression of E-cadherin is facilitated by HDAC3 expression that enhances cellular migration which is essential in metastasis of malignant ovarian cancer (Smith et al., 2017). OX-40 L and 4-1BBL are responsible for regulating the activity of effector cytotoxic T-cells whereas immunosuppressive effects are exhibited by PD-L1(Programmed death ligand 1), protecting the tumor cells to form immune destruction (Takai et al., 2007). HDAC 1 and 3 enhance suppression of OX-40 L and 4-1BBL in chemotherapy-resistant ovarian cancer cells. Additionally, HDAC3 has a role in inflammation whereas HDAC4 facilitates proliferation, invasion potential and migration of ovarian cancer cells by suppressing p21, while HDAC6 is over-expressed in ARID1A-mutated EOCs. Intriguingly, HDAC1 and 7 are over-expressed in cancer stem cells, whereas, HDAC9 and 10 are both essential for homologous recombination in malignant ovarian tumors (Yang et al., 2018). The role HDACi in ovarian cancer is depicted in Figure 4.

### Pre-clinical studies on HDACi in ovarian cancer

Trichostatin A (TSA) is under preclinical stage for ovarian cancer treatment. It has been investigated as a potent anti-tumor agent in a variety of cancers, including ovarian cancer, inhibits both HDACs 1 and 2, induces gene expression of P73 as well as induces Bax-dependent apoptosis (Yano et al., 2018). Additionally, TSA is also involved in the inhibition of VEGF-induced expression of VEGF receptors Nrp1, VEGFR1 and VEGFR2. Moreover, TSA and vorinostat regulate the SEMA3 (VEGF protein competitor) expression, at both mRNA and protein levels (Takai and Narahara, 2010). Whereas, valproic acid (VPA), owing to its anti-angiogenic property, induces down regulation of endothelial nitric oxide synthase (eNOS) in endothelial cells (Eckschlager et al., 2017). The combination of VPA, 5- Azacytidine and carboplatin has been studied by Falchook et al. (2013), but it showed high toxicity. Various cell culture models elucidated that the exposure to VPA results in dose-dependent cell cycle arrest as well as apoptosis in ovarian cancer cell lines (Takai and Narahara, 2010). The ability of VPA to inhibit the growth of human SK-OV-3 ovarian cancer tumors was tested in immunodeficient mice and it was observed that VPA suppresses the tumor growth remarkably along with no observable adverse effects. The studies demonstrated by Qian et al. (2012) revealed that PXD101 in combination with carboplatin cause enhancement in the anti-tumor activity tested on human A2780 ovarian xenografts whereas, paclitaxel in combination vorinostat enhance the anticancer effect as compared to vorinostat as a single agent (Hontecillas-Prieto et al., 2020).

Vorinostatis an HDACi, which has been successfully tested against EOCs (Smith et al., 2017). In clinical trials, vorinostat appeared to be one of the most promising HDACi in the treatment of EOC, as compared to all other HDACi. It proves to be effective in treating ovarian cancer, either individually or in combination with anti-cancer drugs like cisplatin (Moufarrij et al., 2019). About 6 ovarian cancer cell lines were tested against TSA, vorinostat, VPA and NaB, it was found that all the cell lines were sensitive to the drugs, it was observed that vorinostat arrests the cell cycle as well as induces apoptosis in the ovarian cancer cell lines (Takai and Narahara, 2010). Further, vorinostat was found to induce apoptosis (caspase- 3 activity) in about half the ovarian cancer cell lines along with few of the primary cancer cells, isolated from stage III EOC patients. However, vorinostat failed to show anti- tumor activity in platinum- resistant EOC cells. The fact that vorinostat enhanced apoptosis as well as reduced viability in a similar fashion as that of paclitaxel in EOC cell lines, was reported by Cooper et al. (2007), however the combination was not significant statistically. CBHA, TSA, scriptaid, vorinostat, sodium butyrate, VPA, PDX101, MS-275, M344 and apicidin, are the HDACi drugs that exhibited anticancer activity as single agents. An approach involving the combination of HDACi with other agents, namely carboplatin, paclitaxel, cisplatin, docetaxel, etc., are being studied for efficacy in ovarian cancer (Khabele, 2014). This multi- targeted approach is to treat ovarian cancer is highly beneficial as it exploits the varied mechanisms of action of HDACi, thereby producing a synergistic effect with the other agents which finally leads to an enhanced anti-tumor activity in ovarian cancer (Hontecillas-Prieto et al., 2020).

**FIGURE 5:**
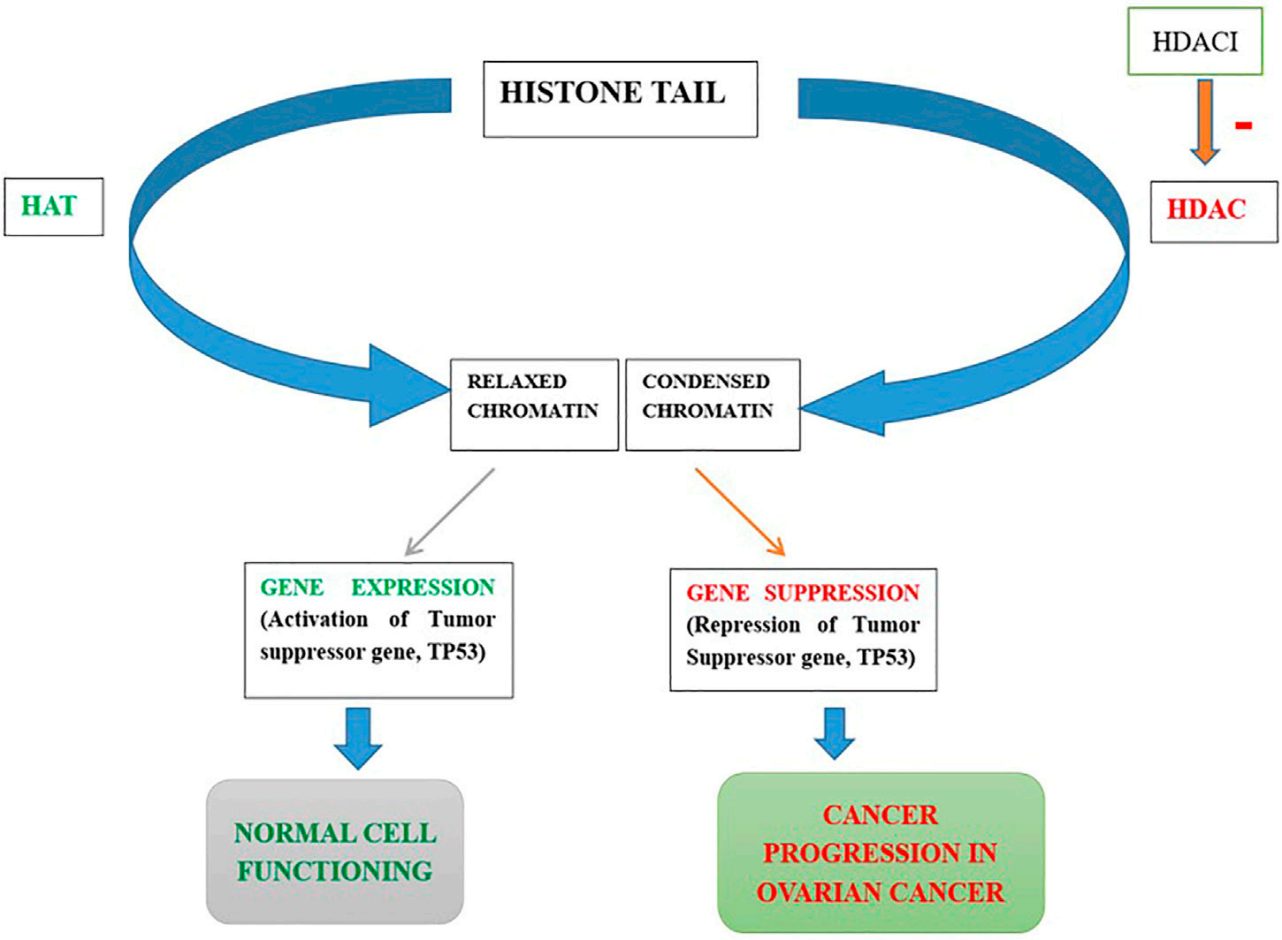
Role of HDACi in treatment ovarian cancer. HDAC, Histone deacetylase; HAT, Histone acetyltransferase; HDACi, Histone deacetylase inhibitor.

On the other hand, it has been stated that scriptaid, apicidin and CBHA enhanced the amount of cells in various phases of the cell cycle (namely the G0/G1 and/or G2/M phases) and reduced the amount of cells in S phase of the cell cycle (Tan et al., 2010). It has been indicated by various studies that apoptosis induced by HDACi is linked to the loss of mitochondrial transmembrane potential along with the altered expression of E-cadherin, p21WAF1, cyclin A, p27KIP1, and p16. It was reported that treatments with scriptaid, apicidin and CBHA enhanced the acetylation of H3 and H4 histone tails (Eckschlager et al., 2017). From these results, it has been observed that the HDACi exert anti-proliferative activity through the induction of selective genes inducing cell growth, malignant phenotype and apoptosis (Takai et al., 2007). Role of HDACi in treatment ovarian cancer is given in Figure 5.

### Registered HDACi in clinical trials of ovarian cancer

Romidepsin, panobinostat as well as vorinostat, are the HDACi that have been successfully tested against ovarian cancer, both individually as well as in combination with cisplatin-like drugs, along with being approved by FDA (Singh et al., 2011). Apart from these three agents, several HDACi are going through rapid development as well as being under investigation in preclinical and clinical trials for having anti-ovarian cancer potential. The impact of belinostat was evaluated in platinum-resistant ovarian cancer population in a study conducted by the Gynecologic Oncology Group (GOG) (Singh et al., 2011). This study was terminated in initial stages, due to high toxicity along with the lack of activity. However, this study was initiated again and phase Ib/II study was performed by Dizon et al. (2012), with an investigative expansion of phase 2 scheduled for women having recurrent EOC for the clinical activity evaluation of BelCaP (Belinostat, Carboplatin and Paclitaxel). It was revealed by the results that 46% of the tested population had primary platinum-resistant disease. On the other hand about 54% of the patients showed recurrence within 6 months of the treatment. BelCaP was administered for 6 (range, 1–23) median no. cycles with an overall response rate of 43% (95% confidence interval, 26%–61%). However, it was observed that median overall survival rate was not attained in the duration of the follow-up study (4 months-median follow-up). It was further revealed from the results that BelCaP was tolerated reasonably well and its clinical benefits were verified in heavily-pretreated EOC patients. The addition of belinostat to this platinum-based regimen represents a novel approach for the therapy of epithelial ovarian cancer (EOC) and required further investigation (Singh et al., 2011).

HDACi like trapoxin have poor bioavailability *in-vivo* along with having adverse effects at high doses and thereby have limited therapeutic use. Phase I clinical trial of phenylbutyrate sodium for dose escalation to test its efficacy in advanced-stage tumor patients was by Camacho et al. (2016) at Memorial Sloan-Kettering Cancer Center. It was observed that the administration of phenylbutyrate sodium twice a day as an infusion schedule was deemed safe, with a maximum tolerated dose of 300 mg/kg/day (Singh et al., 2011). NaB, on the other hand, had a noteworthy suppressive effect on growth in human ovarian cancer cells, regardless of their p53 gene status, as indicated by the study conducted by Terao et al. (2001), it has been studied at length for its antitumor activity, it has been found that NaB can induce cancer cell differentiation, however, its therapeutic potential has been limited by a plasma half-life of only 5 min (short t_1/2_). Vorinostat was subjected to a phase II clinical trial to evaluate its efficacy and toxicity in recurrent or persistent EOC patients. An oral daily dose of 400 mg of vorinostat was continued for a duration of about 3 weeks and toxic side effects barred further therapy with vorinostat (Hontecillas-Prieto et al., 2020). Another drug romidepsin, obtained from a bacterial source *C. violaceum*, is an HDACi and brings about apoptosis in tumor cells. It was approved by FDA in 2009, along with the clinical trials that have been executed in a variety of tumors including ovarian cancer, prostate cancer, breast cancer, etc. (Smith et al., 2017). Many of the other compounds namely abexinostat (PCI24781), givinostat (ITF2357), quisinostat (JNJ-26481585), and resminostat (4SC201) have been investigated recently as the pan-HDACi in clinical trails. However, vorinostat and romidepsin have not been exhibited in studies with various solid tumors including ovarian, cervical tumors, etc. Thus, a combination of various HDACi with other agents are under investigation in clinical trials in order to boost their anticancer potential (Khabele, 2014).

Vorinostat, in combination with gemcitabine and carboplatin along with the continuation of vorinostat, is used in the treatment of platinum-sensitive, recurrent epithelial ovarian, fallopian tube, or peritoneal cancer (Eckschlager et al., 2017). This study was terminated in the IB/II phase of clinical trials due to unacceptable toxicity. Similarly, vorinostat is used to treat primary advanced stage ovarian cancer in combination with paclitaxel and carboplatin. This study was also terminated in phase I/II of the clinical trials, due to unavoidable toxicity like GI perforation with results indicating a complete response of 39%, partial response of 11.2%, and an overall response rate of 50% in the duration of the study (Khabele, 2014).

On the other hand, belinostat (PXD101) combined with carboplatin is used to treat recurrent or persistent platinum-resistant ovarian, fallopian tube, or primary peritoneal cancer. This study was terminated in phase II due to minimal activity and the results show that there was a partial response of 3.7%, 44.4% of stable disease and 29.6% of progressive diseases (Khabele 2014). Similarly, belinostat in combination with carboplatin and paclitaxel is used to treat ovarian cancer, the study was completed in phase I/II with no observed grade 4 toxicities, the results indicated a complete response of 8.6%, 34.2% of partial response, and an overall response rate of 43% on completion of the study. A minor response was obtained in phase I of clinical trial of valproic acid, in combination with 5-azacytidine and carboplatin, in treatment of platinum resistant epithelial ovarian cancer (Smith et al., 2017). Recent HDACi in clinical trials of ovarian cancer have been shown in Table 7. Also the role of HDACi in combination with other drugs in clinical research and the status of clinical research on HDACIs have been shown in Table 8 and Table 9, respectively.

**TABLE 7:** Recent HDACi in clinical trials of ovarian cancer.

SI no.	HDACi	Drugs in combination	Phase of clinical trial	Type of cancer	References
01	Tinostamustine (EDO-S101)	Capecitabine	Phase I and II	Ovarian cancer	NCT03345485 Kumar et al. (2017)
02	Belinostat	Talazoparib	Phase I	Metastatic Ovarian Carcinoma	NCT04703920 Ramarao-Milne et al. (2021)

**TABLE 8:** Role of HDACi in combination with other drugs in clinical research.

HDACi	Drugs in combination	Target of treatment	Phase of clinical trial	References
Vorinostat	Gemcitabine and carboplatin along with continuation of vorinostat	Platinum-sensitive, recurrent epithelial ovarian, fallopian tube, or peritoneal cancer	Phase IB/II [terminated due to unacceptable toxicity]	NCT00910000 Matulonis et al. (2015)
Vorinostat	Paclitaxel and carboplatin	Primary advanced stage ovarian cancer	Phase I/II [terminated due to unacceptable toxicity, like GI perforation] 39% complete response, 11.2% partial response and 50% overall response rate	NCT00976183 Mendivil et al. (2013)
Belinostat	Carboplatin	Recurrent or persistent platinum-resistant ovarian, fallopian tube, or primary peritoneal cancer	Phase II Terminated due to minimal activity 3.7% partial response 44.4% stable disease 29.6% progressive disease	NCT00993616 Dizon et al. (2012)
Belinostat	Carboplatin and paclitaxel	Ovarian cancer	Phase I/II completed No toxicities of grade 4 8.6% complete response 34.2% partial response 43% overall response rate	NCT00421889 Dizon et al. (2012)
Valproic Acid (VPA)	5-Azacytidine and carboplatin	Platinum-resistant EOC	Phase I minor response or stable disease	Falchook et al. (2013)

**TABLE 9:** Status of clinical research on HDACi.

Sl.No.	Drug	Combination	Phase of trial	Disease targeted	References
1	Tinostamustine (EDO-S101)	Capecitabine	Phase I & II	Ovarian cancer	NCT03345485 Kumar et al. (2017)
2	Belinostat	Talazoparib	Phase I	Metastatic ovarian carcinoma	NCT04703920 Ramarao-Milne et al. (2021)
3	Panobinostat	Bortezomib & dexamethozone carfilzomib	Phase III	Multiple myeloma (increase in progression free survival)	NCT01023308 Richardson et al. (2016)
			Phase II	Multiple myeloma (combination safe and effective)	NCT01549431 Kaufman et al. (2019)
4	MPT0G413	Bortezomib	Preclinical	Multiple myeloma (reduced tumor cell viability and growth)	Huang et al. (2019)
5	Nexturastat	A5-Azacytidine	Preclinical	Ovarian cancer	Moufarrij et al. (2020)
6	Vorinostat	Carfilzomib	PhaseI	B-cell lymphomas	NCT01276717 Holkova et al. (2016), Hontecillas-Prieto et al. (2020)
7	Entinostat	Aldesleukin	PhaseI/II	Renal cell carcinoma	NCT01038778
8	Citarinostat (ACY-241)	Pomalidomide	Preclinical	Mutliple myeloma	North et al. (2017)
9	Ricolinostat (ACY-1215)	Bendamustine	Preclinical	Lymphoma	Cosenza et al. (2017)
		Oxaliplatin	Preclinical	Colorectal cancer	Lee et al. (2019)
		Bortezomib and Dexamethasone	Preclinical	Multiple myeloma	Vogl et al. (2017)
		Carfilzomib	Preclinical	Multiple myeloma	Mishima et al. (2015)
		Bortezomib	PhaseI/II	Multiple myeloma	NCT01323751
		Ibrutinib	Preclinical	Lymphoma	Amengual et al. (2015)
		Lenalidomide and dexamethasone	PhaseIb	Multiple myeloma	NCT01583283 Yee et al. (2016)
10	Romidepsin	Erlotinib	PhaseI	Non-small cell lung cancer	NCT01302808 Gerber et al. (2015)

## Conclusion and future direction

HDACs participates in a variety of physiological processes of cells through histone and non-histone substrates, and is closely related to the occurrence and development of cancer. HDACi can inhibit the proliferation of cancer cells and promote apoptosis, and have become an effective means of cancer treatment. Over the last decade, numerous HDACi have been discovered with five of them (Vorinostat, FK-288, PXD-101, panobinostat and CS-055) approved for clinical use as anticancer drugs. However, there are two major problems that limit the clinical uses of HDACi, which include toxicity and drug resistance. The toxicity of current pan-HDACi is largely attributed to the lack of HDACs isoforms selectivity; while the causes of drug resistance to HDACi are multi-fold, including but not limiting to the (re) activation of other signaling pathways such as CDK and AKT.
